# Goldilocks and Entrustment: Finding the Amount of Learner Autonomy That's Just Right

**DOI:** 10.15766/mep_2374-8265.10987

**Published:** 2020-10-13

**Authors:** Kelly Skelly, Parang Kim, Marcy Rosenbaum, Jason Wilbur

**Affiliations:** 1 Associate Professor, Department of Family Medicine, University of Iowa Carver College of Medicine; 2 Research Assistant, Department of Family Medicine, University of Iowa Carver College of Medicine; 3 Professor, Department of Family Medicine, University of Iowa Carver College of Medicine

**Keywords:** Autonomy, Professional Autonomy, Entrustment, Feedback, Communication

## Abstract

**Introduction:**

Faculty and residents strive for appropriate autonomy and entrustment. Initial direct supervision of clinical care gradually shifts to increasing levels of resident independence over time. Faculty members are inconsistent in resident supervision leading to missed opportunities for resident independence.

**Methods:**

Family medicine faculty workshop participants completed teaching style self-evaluations prior to discussion of clinical examples with excessive or insufficient autonomy. Participants reviewed real resident feedback examples to increase insight into teaching styles. Participants were presented with cases to discuss varying degrees of resident autonomy and entrustment. Learners committed to one specific behavior to calibrate the degree of autonomy they provide.

**Results:**

Of the faculty, 113 members participated in the workshop with the majority (98%) finding the workshop relevant in helping them to identify strategies for reflecting on their degree of autonomy allowed and to look for appropriate situations for enhancing their resident entrustment.

**Discussion:**

This interactive workshop provided clear ways for addressing the issue of independence versus control in supervision of patient care. It provided a feedback mechanism for educators who provide too much or too little autonomy for the best resident learning. Additionally, this conversation encouraged participants to engage in self-reflection on the autonomy given to their resident.

## Educational Objectives

By the end of this activity, faculty participants will be able to:

1. Describe the potential impact of how the degree of autonomy and entrustment given to the resident physicians influences their view of faculty teaching.
2. Discern when greater/lesser autonomy is required for learning and patient safety by analyzing scenarios with excessive or insufficient autonomy granted to resident physicians.
3. Evaluate their own individual faculty personal teaching styles to recognize how they impact autonomy.
4. Apply at least one specific behavior to appropriately calibrate the degree of autonomy provided to resident physician in clinical situations.

## Introduction

According to the 2019 ACGME Common Program Requirements, residency is a crucial time of professional development as resident physicians transition from students to autonomous clinicians.^1^ As a result, all faculty and residents strive to provide the appropriate amount of autonomy and entrustment.^2–10^ Though there are many articles on clinical teaching where there is implicit information on autonomy and learners,^11^ and Carbo provides a toolkit to teach learners autonomy,^12^ little is published that provides explicit strategies for assessing learner readiness for independent practice and supporting resident autonomy.^13^

Family medicine residents are expected to progressively develop the ability to independently care for patients over the course of their 3-year residency. Initial direct supervision of all aspects of care gradually shifts to increasing levels of resident independence over time. At various points during this evolution, residents may experience too much or too little autonomy from supervising faculty. Faculty describe fear of malpractice, inexperience, and practice style as reasons they may be less likely to allow autonomy to resident learners.^14^ Resident complaints about the degree of independence they are granted, either too much or too little, fail to consider whether there are resident issues at play such as where the resident is inexperienced, or has not demonstrated evidence of good clinical decision-making.^13^ Additionally, the resident concerns do not consider the environment and patient factors affecting the entrustment issues.^14^

Faculty members may not always be able to judge how much resident autonomy is appropriate or desired, leading to frustration on the part of more senior residents and potentially insufficient supervision for more junior residents. Additionally, faculty may not have insight into the degree of autonomy they allow and whether it is appropriate for the level of learner. Currently, *MedEdPORTAL* has only one resource related to this topic of autonomy, a computer program allowing learner autonomy in clinical situations which did not provide curricula where faculty can self-evaluate and address the amount of learner autonomy allowed.^15^ This workshop will not provide skills to provide graduated entrustment based on entrustable professional activities, but will provide a means to focus on faculty factors and faculty styles in an attempt to address and calibrate the faculty role in autonomy.

## Methods

### Workshop Development

This interactive workshop offered insight about how a mismatch between guidance provided and resident perceived needs can impact the learning environment. Utilizing Kern's curriculum development approach, the workshop addressed faculty affective objectives to result in faculty attitudinal changes about giving independence to residents.^16^ Kern's approach gave the ability to provide congruence between our affective objectives and educational methods; the workshop's instructional approach worked well with this because it utilized real-life experience examples, group discussion, and reflection.^16^ It provided exercises for addressing the issue of independence versus control in the supervision of patient care and the need for feedback to be given to educators who provide too much or too little autonomy for the best resident learning. The workshop was given as a required departmental faculty development conference as part of a series of workshops to all faculty teachers, and at a national meeting for family medicine residency program faculty. Our target audience in both cases were faculty members and educators involved in clinical medical education. This workshop was given to family medicine educators, but would be easily translated to any specialty with this educational resource.

### Setup

The session took 1 hour and required a room with a computer and projector. The PowerPoint slides (Appendix A), self-evaluation activity (Appendix B), small-group exercise 1 (Appendix C), small-group exercise 2 with comment evaluation activity (Appendix D), cases (Appendices E and F), audience commitment form (Appendix G), and postworkshop evaluation (Appendix H) are available with a facilitator's guide (Appendix I) describing the process. For ease of printing materials prior to the workshop, Appendix J clearly provided all participant materials. The workshop was completed with variable group sizes but can be completed with groups as small as five to six participants and up to 100 participants due to the frequent small-group activities allowing individual participants to benefit from discussion; with less than five participants, the outcomes of the discussions might be less effective.

### Process

Prior to the workshop, the Grasha-Reichmann Teaching Style Inventory^17,18^ scale was provided via email for participants to assess their own teaching style, but this is optional. The workshop process, which was provided in detail in the facilitator's guide (Appendix I), was as follows:

#### 1 minute

Upon arrival, participants were asked to do a self-evaluation of the degree of autonomy they allowed learners (Appendix B). This activity was for their own reflection.

#### 5 minutes (small-group exercise 1—reflection)

Participants divided into small groups and came up with examples of educators they had met in their training and career who were too controlling or too laid back (Appendix C). Volunteer participants shared a few examples of what the small group brainstormed with the entire group. The purpose of the exercise was to get participants thinking of both sides of the continuum and recognizing that either too much or too little autonomy would be possible.

#### 2 minutes

Workshop objectives were reviewed (Appendix A, slide 2).

#### 10 minutes (small-group exercise 2—comment evaluation activity)

We distributed hard copies of anonymized comments from resident evaluations of faculty supervision to each small group (Appendix D). These comments were collected throughout the year from the authors' residency evaluations of faculty. The groups took the time to alternate reading the comments out loud and determining whether they thought the comments were positive or negative. Additionally, participants were prompted to consider whether the comments could have been made about them as a faculty member. This was to help provide insight into the situation for those who have less insight than they might need.

#### 1 minute

The faculty participants completed the self-evaluation a second time (Appendix B). They were instructed to reflect on any changes in their self-evaluation since the beginning of the workshop.

#### 10 minutes

Case 1 (Appendix E) presented a resident case to allow discussion about the appropriate amount of faculty involvement in the resident's patient care. This case was reviewed with discussion about how faculty could react with too much or too little autonomy and what cues the resident might provide to impact this.

#### 5 minutes

A discussion of teaching and learning styles based on the Grasha-Reichmann Teaching Style Inventory (optional activity) and teaching and learning styles for participants (PowerPoint slides) was completed.^17,18^ This information highlighted the faculty physician opportunities to impact resident physician learner autonomy further by considering the actual resident physician learning style prior to the second case discussion and was optional.

#### 10 minutes

Case 2 (Appendix F) presented a second resident case to allow discussion about the appropriate amount of faculty involvement in the resident's patient care. This case was again reviewed with discussion about how faculty could react with too much or too little autonomy and what cues the resident might provide to impact this.

#### 5 minutes

The final exercise was to have each attendee commit to one thing to change, one thing to continue, and one thing to add to teaching. We asked participants to write these on an index card and emailed them this information 3 weeks later to remind them to follow-up on this learning (Appendix G).

#### 1 minute

Evaluations of the workshops were completed at all the workshop presentations (Appendix H). Participants were asked to evaluate 5 questions about the session on a 5-point Likert scale (1 = *not at all,* 5 = *extremely*).

### Evaluation Across Settings

We evaluated whether this curriculum was applicable outside the University of Iowa Family Medicine Residency Program when we presented this curriculum at the American Academy of Family Physicians Program Directors Workshop (AAFP PDW).^19^ A modified version of the postworkshop evaluation was administered to this group.

## Results

University of Iowa family medicine faculty who attended the workshop and completed the survey (*n* = 20) suggested that they found the workshop to be useful (*M* = 4.2) and of interest (*M* = 4.4) with a high likelihood that they would apply what they learned (*M* = 4.4; Table 1). Comments suggested that the discussion was engaging and valuable. Faculty commitment forms revealed one specific behavior each faculty would adopt to calibrate the degree of autonomy they provided to residents in clinical situations for the future (Table 2). Attendees were all emailed their commitment to change as a reminder 3 weeks after the workshop.

**Table 1.**
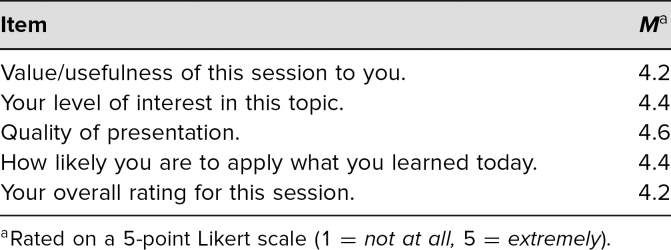
Postworkshop Evaluation Responses from Faculty Development Autonomy Workshop (*n* = 20)

**Table 2.**
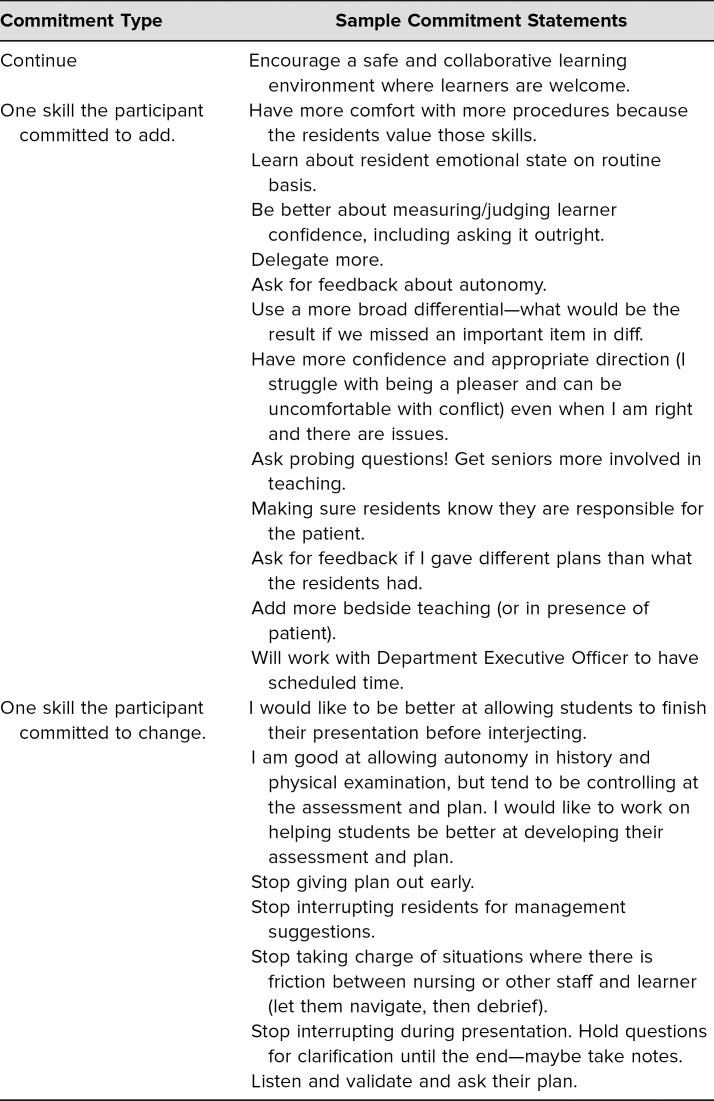
Commitment Examples from Faculty Development Autonomy Workshop

At the AAFP PDW, 63 participants evaluated the workshop with the standard AAFP PDW survey and most participants (98%) felt the topic was of high value and relevant to training programs. We did not collect commitments to change or continue or add to teaching at this setting because we did not have contact information for the participants. Comments suggested that starting with looking at de-identified comments from residents by faculty would be of significant interest.

## Discussion

There is a wide range of teaching styles among faculty members, some of which residents consider too controlling while others may be too lax. Sharing comments and discussing scenarios led to some general agreement among faculty members as to what amounts of supervision and autonomy are appropriate in different situations. We found that opportunities could exist to impact autonomy and entrustment using self-reflection and sharing of experiences among faculty members. Additionally, we found that sharing resident feedback of faculty members (in a redacted and therefore safe format), allowed faculty insight into residents' experiences of autonomy and entrustment while under their supervision.

### Limitations

This workshop utilized several of many available tools to highlight the opportunities to impact teaching styles. While the Grasha-Reichmann Teaching Style Inventory utilized here was functional,^17,18^ it is also possible to utilize a newer tool from medical teaching or clinical settings in the future which could address personality, learning, or other individualized participant needs as determined by the facilitators. The self-evaluation scale could be adjusted to allow for less bias if it was on a smaller scale than 10 points, and could have different words or no words to decrease bias regarding the person completing the scale. However, because it was a self-reflection tool, the absolute number did not carry much significance because it was relative to the self-reflector and helped to increase self-reflection and insight into teaching styles.

Though the attached sample comments could be used (Appendix D), it might be better to obtain and use anonymized comments from the users' residency program. These comments provided in Appendix D are specific to family medicine residents and would be best utilized in workshops for educators in this specialty. By providing specialty-specific comments, the comments had potential to uncover issues related to a specific specialty residency that could be addressed.

This workshop only has two case studies. Using the case studies may help faculty recognize when more or less autonomy is warranted, and possibly additional case studies with more or less clear resident experience or patient complexity could be useful to encourage discussion for situations that could encourage or discourage faculty autonomy allowed. Additionally, case studies specific to the specialty of the faculty educator will likely be most effective and would need to be created for other specialties to utilize this curriculum.

### Future Directions

The comment evaluation activity (Appendix D) had potential to take too much time from learning how to determine appropriate level of resident autonomy. However, attendees stated that they rarely saw resident comments of other faculty's teaching, suggesting that resident comments have the potential to provide insight into individual faculty behaviors that might not have been recognized otherwise. To better capture whether faculty benefit from reading comments directed toward other faculty, future research could investigate the relationship between resident's comments and faculty's insight or lack thereof about the right amount of resident independence they allow. Currently, the comment review exercise allows participants to recognize resident responses to various faculty behaviors. Comments could be reviewed prior to the workshop to save time if desired, but discussion would be lacking and perhaps make the interaction less valuable.

While this workshop may help the educator gain insight into the degree of autonomy and entrustment they allow learners and may provide initial ideas about assessing learner style, the single workshop did not provide the full skill set necessary to assess competence and appropriate level of autonomy. The time limitation allowed focus on faculty entrustment behaviors and biases, but did not allow the time to delve into specific resident behaviors or detailed patient scenarios. The workshop may be more effective if it was combined with a second session addressing this to provide some insight into how to assess these learners in real-time clinical settings and provide some guidance for how to right-size the amount of autonomy allowed by assessing specific resident behaviors.^20^ A second workshop may be more effective if the faculty have completed this curriculum to self-reflect and effectively remove their own biases to allow resident and patient factors to be addressed.

### Conclusion

This workshop was a unique approach to allow participating faculty to self-reflect on their teaching styles, recognize situations where they did not allow the resident appropriate levels of trust or autonomy, and identify where they could improve their own teaching to allow appropriate resident independence. By having participants commit to learning one specific skill, participants can apply the teachings in a specific manner.^21^ Faculty members found the workshop worthwhile and worth repeating.

## Appendices

Goldilocks and Entrustment Workshop.pptxSelf-Evaluation Activity.docxSmall-Group Activity 1-Reflection.docxSmall-Group Activity 2-Comment Evaluation.docxCase 1-Dr. Newby.docxCase 2-Dr. Almostdone.docxAudience Commitment Form.docxPostworkshop Evaluation.docxAutonomy and Entrustment Facilitator Guide.docxAll Autonomy Workshop Handouts.docx
All appendices are peer reviewed as integral parts of the Original Publication.
