# Supplementary material for: Goldilocks and Entrustment: Finding the Amount of Learner Autonomy That's Just Right
Source: MedEdPORTAL. 2020 Oct 13;16:10987. doi: 10.15766/mep_2374-8265.10987 (PMC7566225; doi:10.15766/mep_2374-8265.10987)
Supplement: Supplementary file 1 — Goldilocks and Entrustment Workshop.pptxSelf-Evaluation Activity.docxSmall-Group Activity 1-Reflection.docxSmall-Group Activity 2-Comment Evaluation.docxCase 1-Dr. Newby.docxCase 2-Dr. Almostdone.docxAudience Commitment Form.docxPostworkshop Evaluation.docxAutonomy and Entrustment Facilitator Guide.docxAll Autonomy Workshop Handouts.docx [file mep_2374-8265.10987-s001.zip › A. Goldilocks and Entrustment Workshop.pptx]

## Slide 1
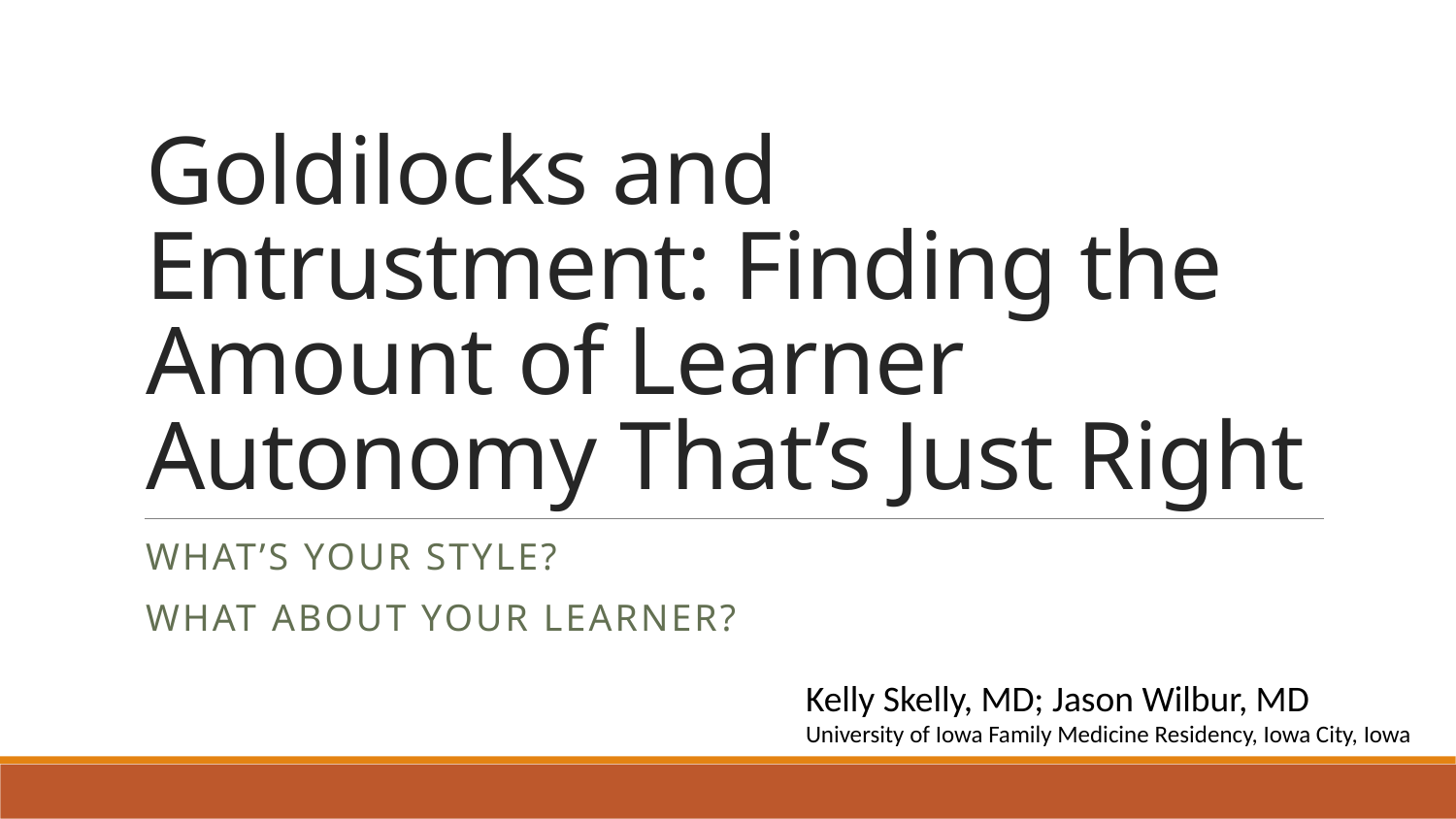

# Goldilocks and Entrustment: Finding the Amount of Learner Autonomy That’s Just Right
What’s your style?
What about your learner?
Kelly Skelly, MD; Jason Wilbur, MD
University of Iowa Family Medicine Residency, Iowa City, Iowa

## Slide 2
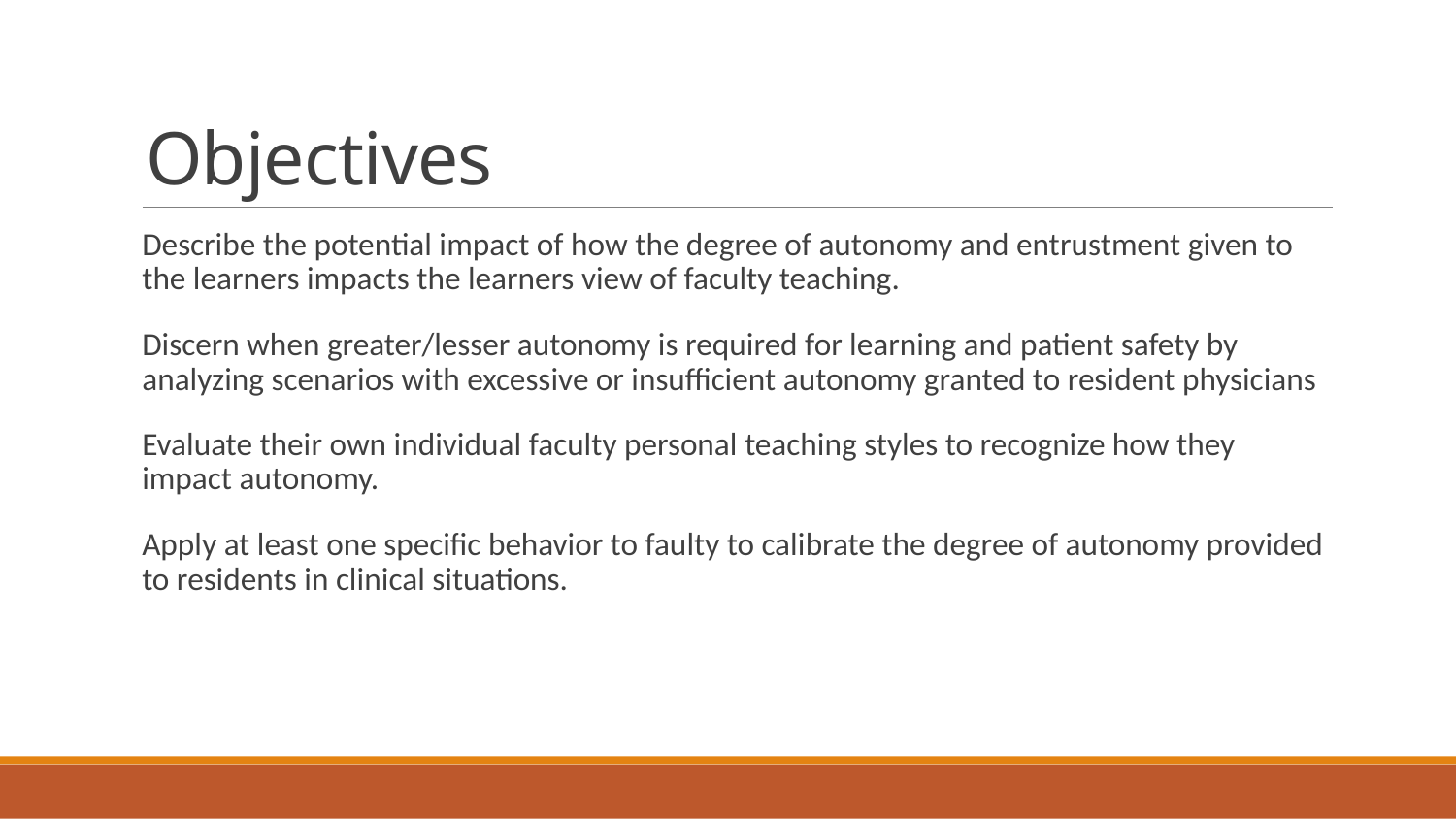

# Objectives
Describe the potential impact of how the degree of autonomy and entrustment given to the learners impacts the learners view of faculty teaching.
Discern when greater/lesser autonomy is required for learning and patient safety by analyzing scenarios with excessive or insufficient autonomy granted to resident physicians
Evaluate their own individual faculty personal teaching styles to recognize how they impact autonomy.
Apply at least one specific behavior to faulty to calibrate the degree of autonomy provided to residents in clinical situations.

## Slide 3
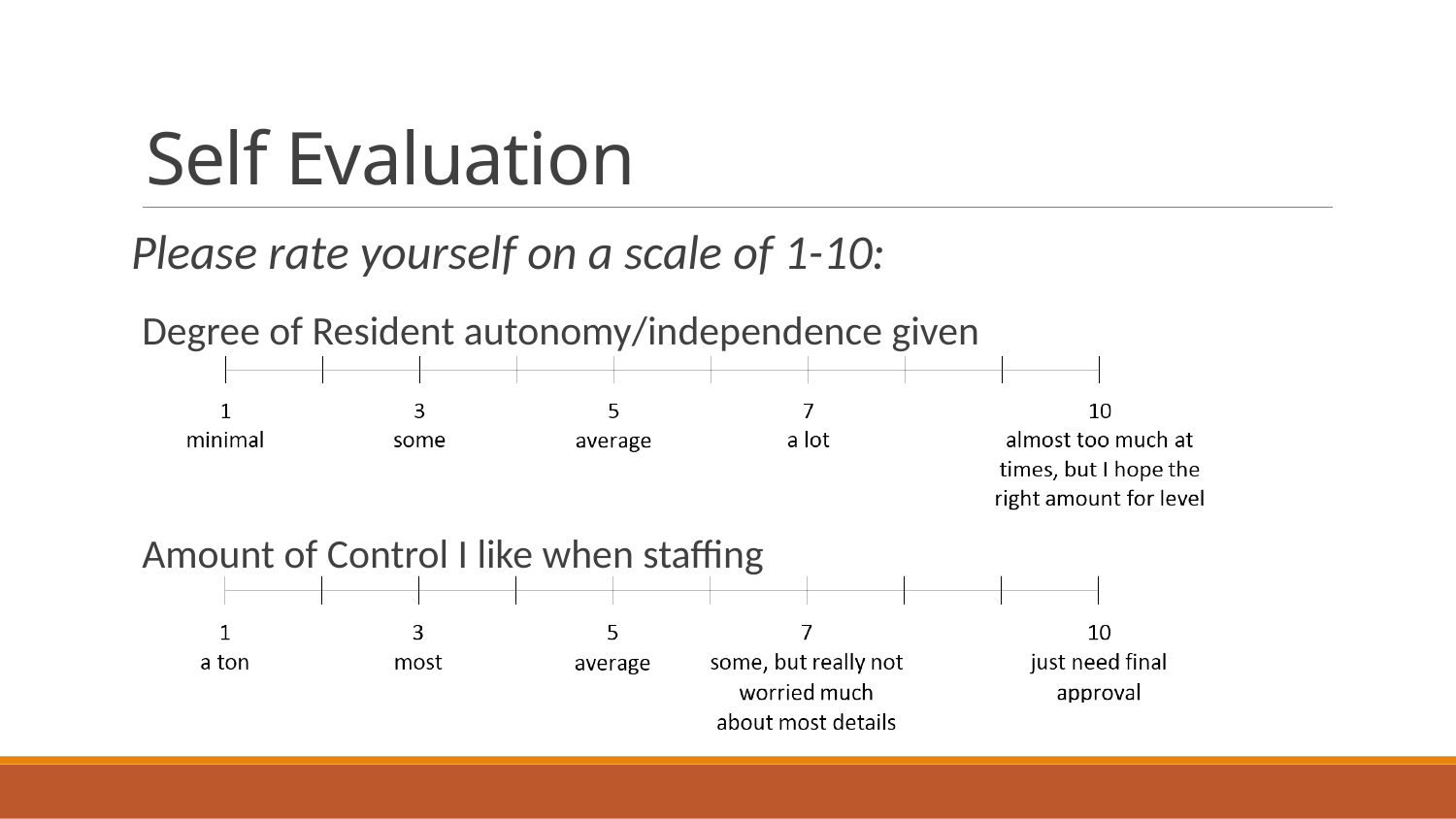

# Self Evaluation
Please rate yourself on a scale of 1-10:
Degree of Resident autonomy/independence given
Amount of Control I like when staffing

## Slide 4
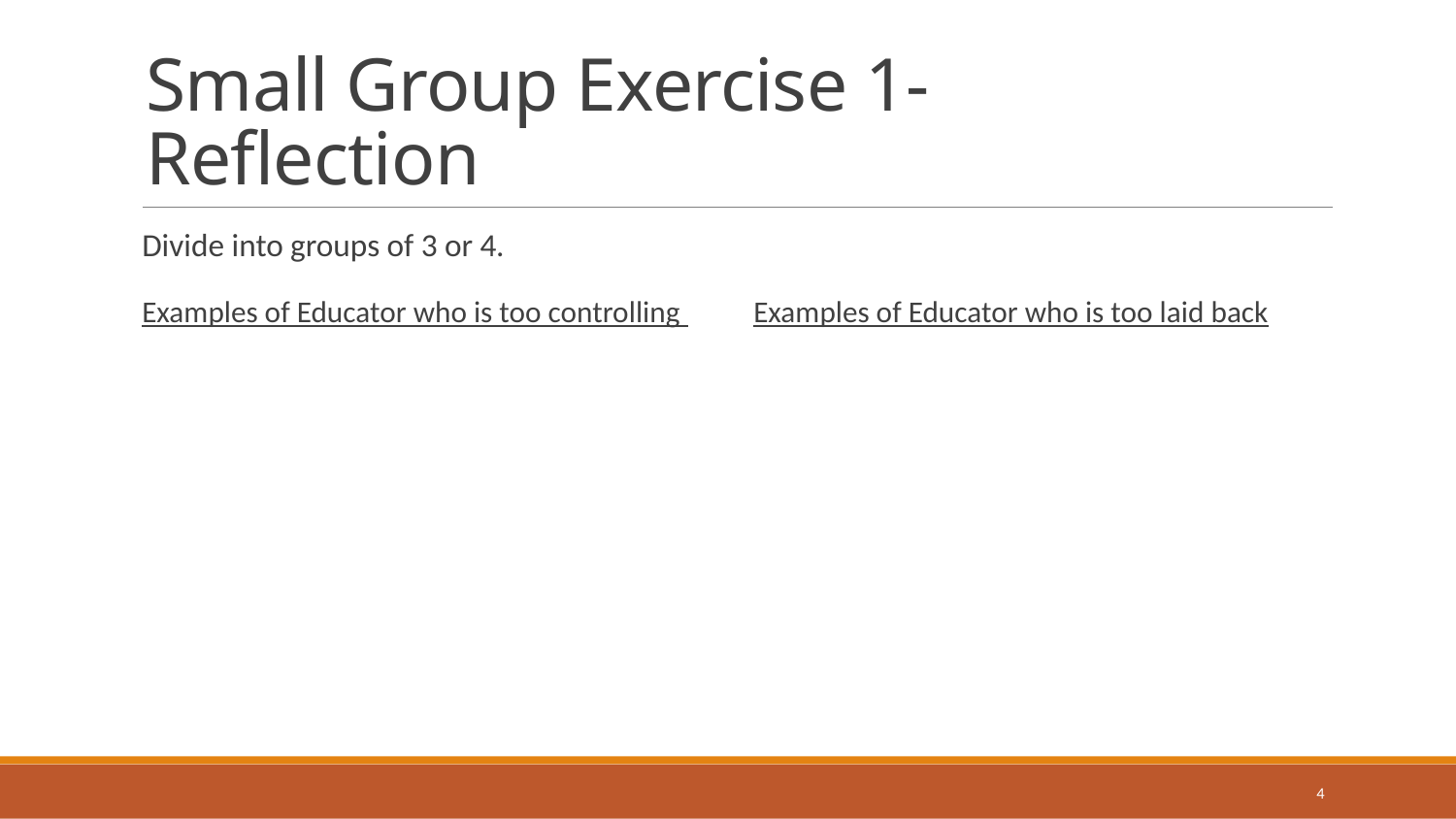

# Small Group Exercise 1- Reflection
Divide into groups of 3 or 4.
Examples of Educator who is too controlling
Examples of Educator who is too laid back
4

## Slide 5
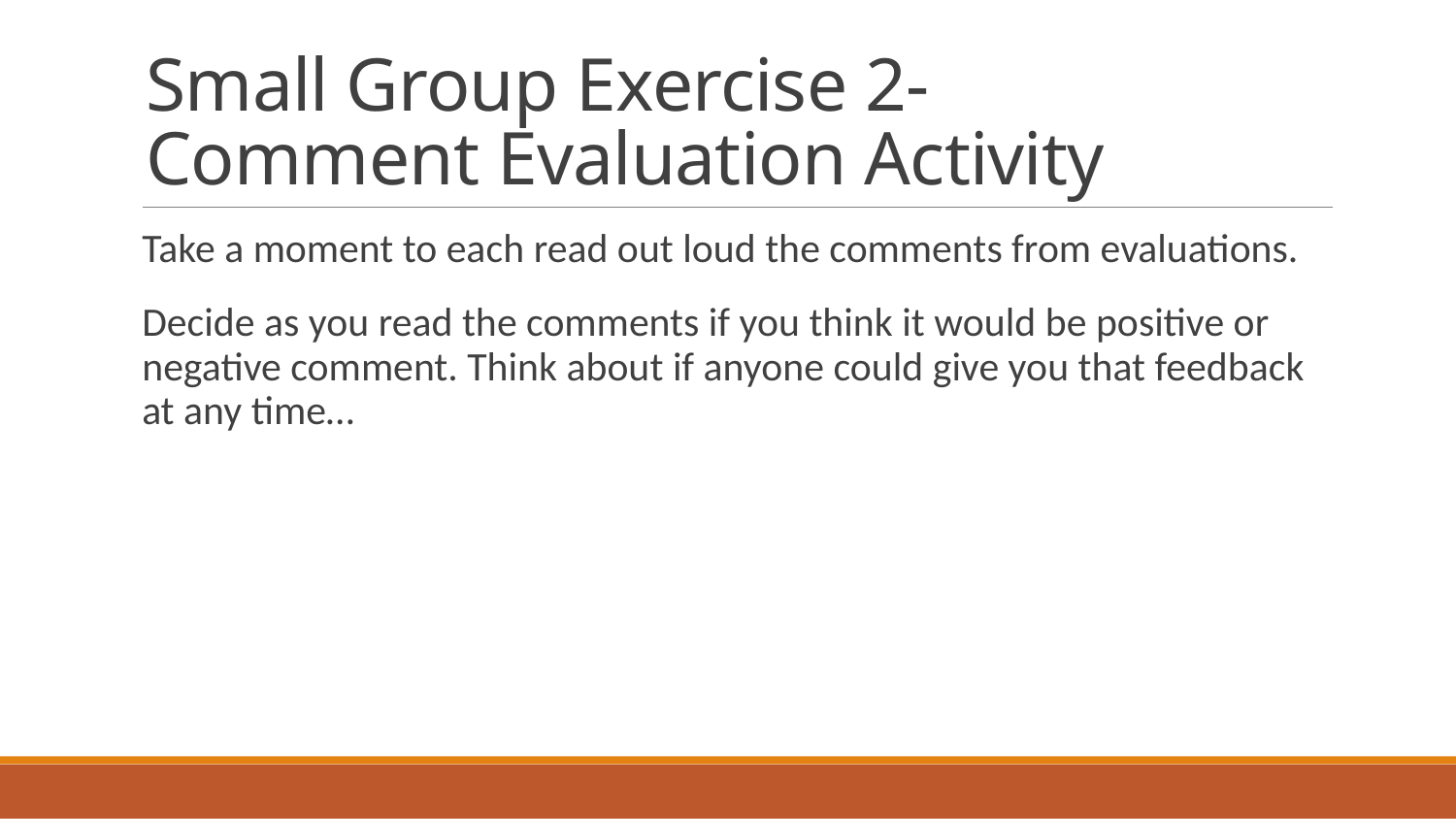

# Small Group Exercise 2- Comment Evaluation Activity
Take a moment to each read out loud the comments from evaluations.
Decide as you read the comments if you think it would be positive or negative comment. Think about if anyone could give you that feedback at any time…

## Slide 6
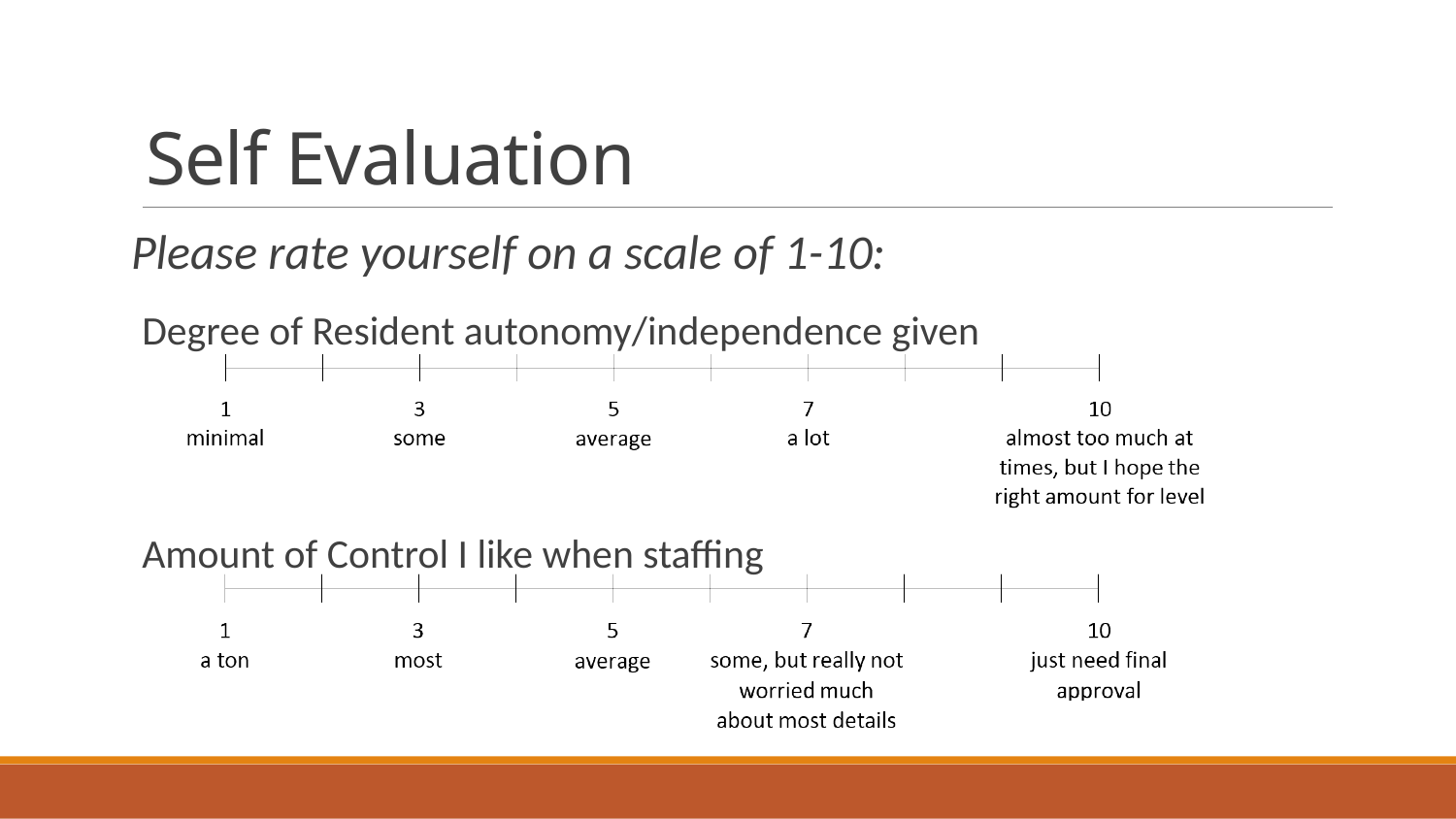

# Self Evaluation
Please rate yourself on a scale of 1-10:
Degree of Resident autonomy/independence given
Amount of Control I like when staffing

## Slide 7
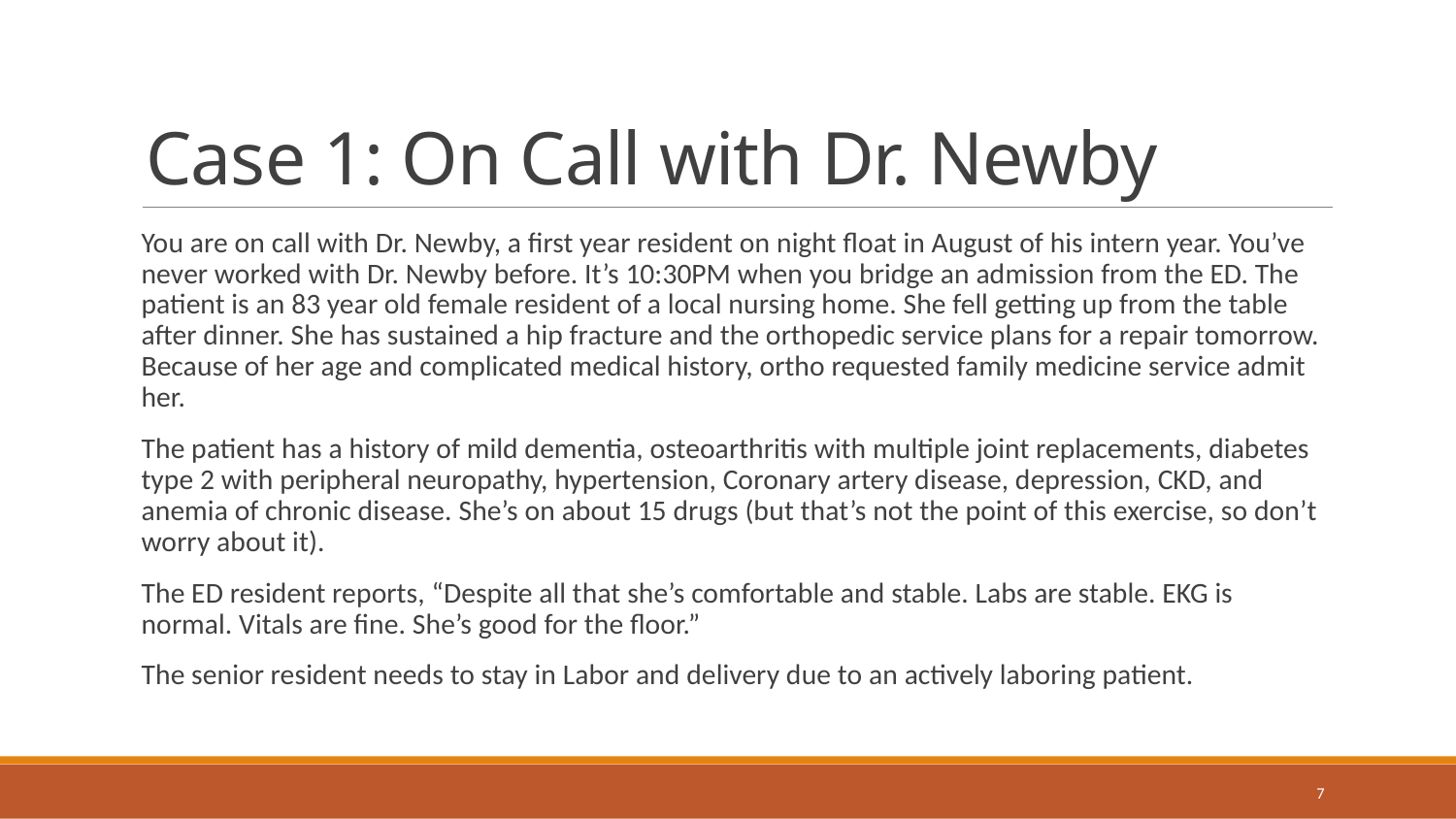

# Case 1: On Call with Dr. Newby
You are on call with Dr. Newby, a first year resident on night float in August of his intern year. You’ve never worked with Dr. Newby before. It’s 10:30PM when you bridge an admission from the ED. The patient is an 83 year old female resident of a local nursing home. She fell getting up from the table after dinner. She has sustained a hip fracture and the orthopedic service plans for a repair tomorrow. Because of her age and complicated medical history, ortho requested family medicine service admit her.
The patient has a history of mild dementia, osteoarthritis with multiple joint replacements, diabetes type 2 with peripheral neuropathy, hypertension, Coronary artery disease, depression, CKD, and anemia of chronic disease. She’s on about 15 drugs (but that’s not the point of this exercise, so don’t worry about it).
The ED resident reports, “Despite all that she’s comfortable and stable. Labs are stable. EKG is normal. Vitals are fine. She’s good for the floor.”
The senior resident needs to stay in Labor and delivery due to an actively laboring patient.
7

## Slide 8
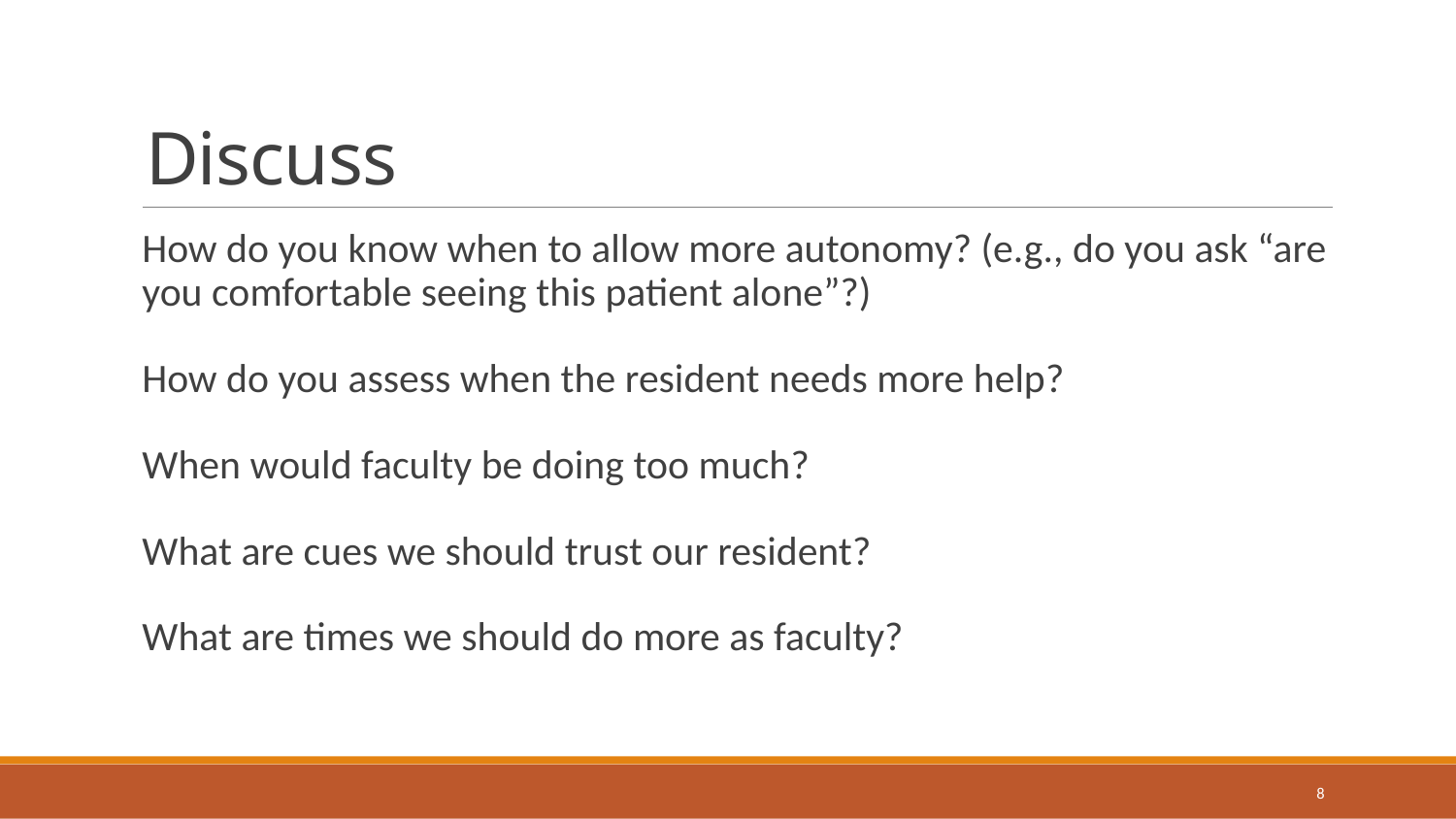

# Discuss
How do you know when to allow more autonomy? (e.g., do you ask “are you comfortable seeing this patient alone”?)
How do you assess when the resident needs more help?
When would faculty be doing too much?
What are cues we should trust our resident?
What are times we should do more as faculty?
8

## Slide 9
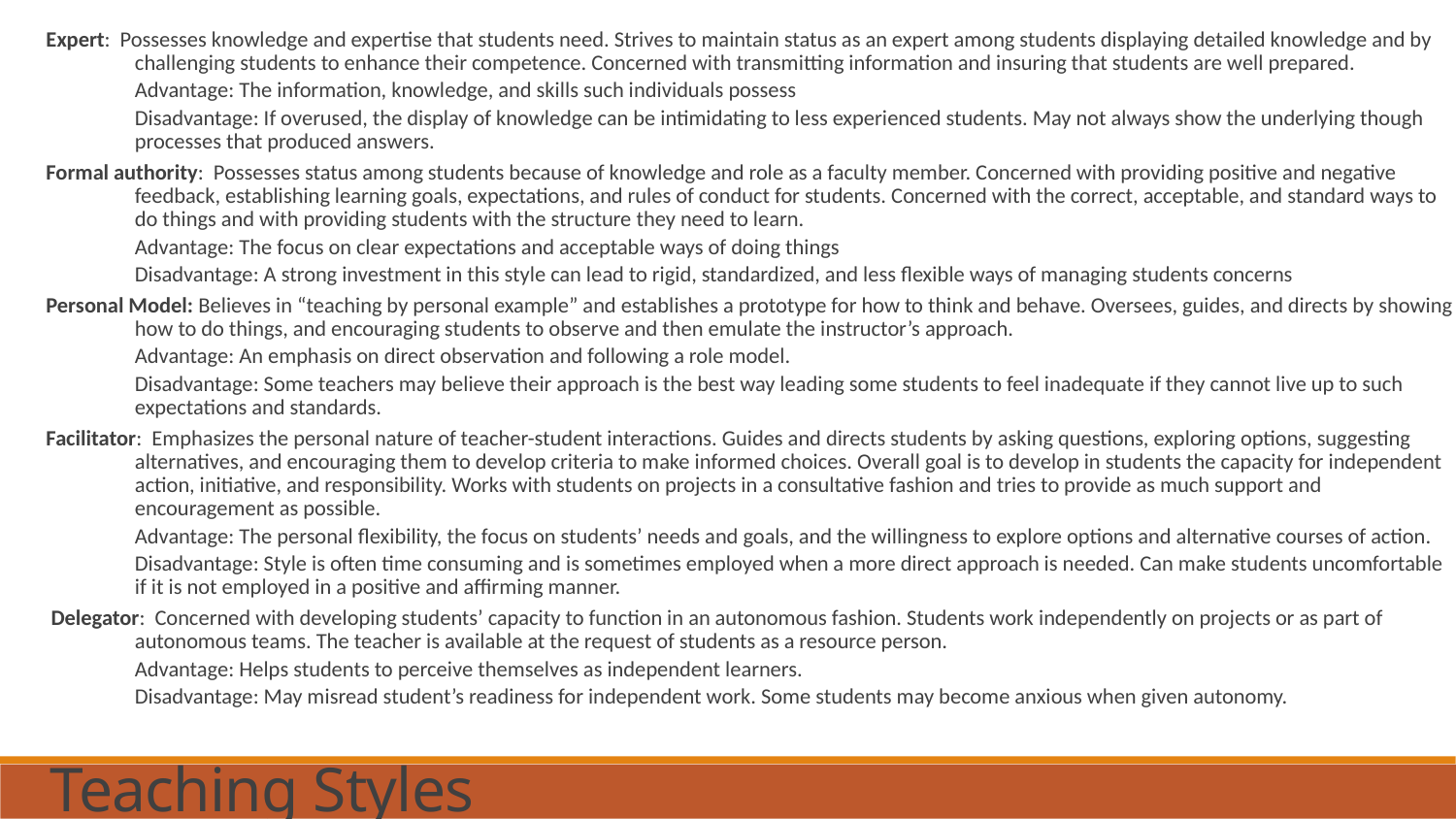

Expert: Possesses knowledge and expertise that students need. Strives to maintain status as an expert among students displaying detailed knowledge and by challenging students to enhance their competence. Concerned with transmitting information and insuring that students are well prepared.
Advantage: The information, knowledge, and skills such individuals possess
Disadvantage: If overused, the display of knowledge can be intimidating to less experienced students. May not always show the underlying though processes that produced answers.
Formal authority: Possesses status among students because of knowledge and role as a faculty member. Concerned with providing positive and negative feedback, establishing learning goals, expectations, and rules of conduct for students. Concerned with the correct, acceptable, and standard ways to do things and with providing students with the structure they need to learn.
Advantage: The focus on clear expectations and acceptable ways of doing things
Disadvantage: A strong investment in this style can lead to rigid, standardized, and less flexible ways of managing students concerns
Personal Model: Believes in “teaching by personal example” and establishes a prototype for how to think and behave. Oversees, guides, and directs by showing how to do things, and encouraging students to observe and then emulate the instructor’s approach.
Advantage: An emphasis on direct observation and following a role model.
Disadvantage: Some teachers may believe their approach is the best way leading some students to feel inadequate if they cannot live up to such expectations and standards.
Facilitator: Emphasizes the personal nature of teacher-student interactions. Guides and directs students by asking questions, exploring options, suggesting alternatives, and encouraging them to develop criteria to make informed choices. Overall goal is to develop in students the capacity for independent action, initiative, and responsibility. Works with students on projects in a consultative fashion and tries to provide as much support and encouragement as possible.
Advantage: The personal flexibility, the focus on students’ needs and goals, and the willingness to explore options and alternative courses of action.
Disadvantage: Style is often time consuming and is sometimes employed when a more direct approach is needed. Can make students uncomfortable if it is not employed in a positive and affirming manner.
 Delegator: Concerned with developing students’ capacity to function in an autonomous fashion. Students work independently on projects or as part of autonomous teams. The teacher is available at the request of students as a resource person.
Advantage: Helps students to perceive themselves as independent learners.
Disadvantage: May misread student’s readiness for independent work. Some students may become anxious when given autonomy.
Teaching Styles

## Slide 10
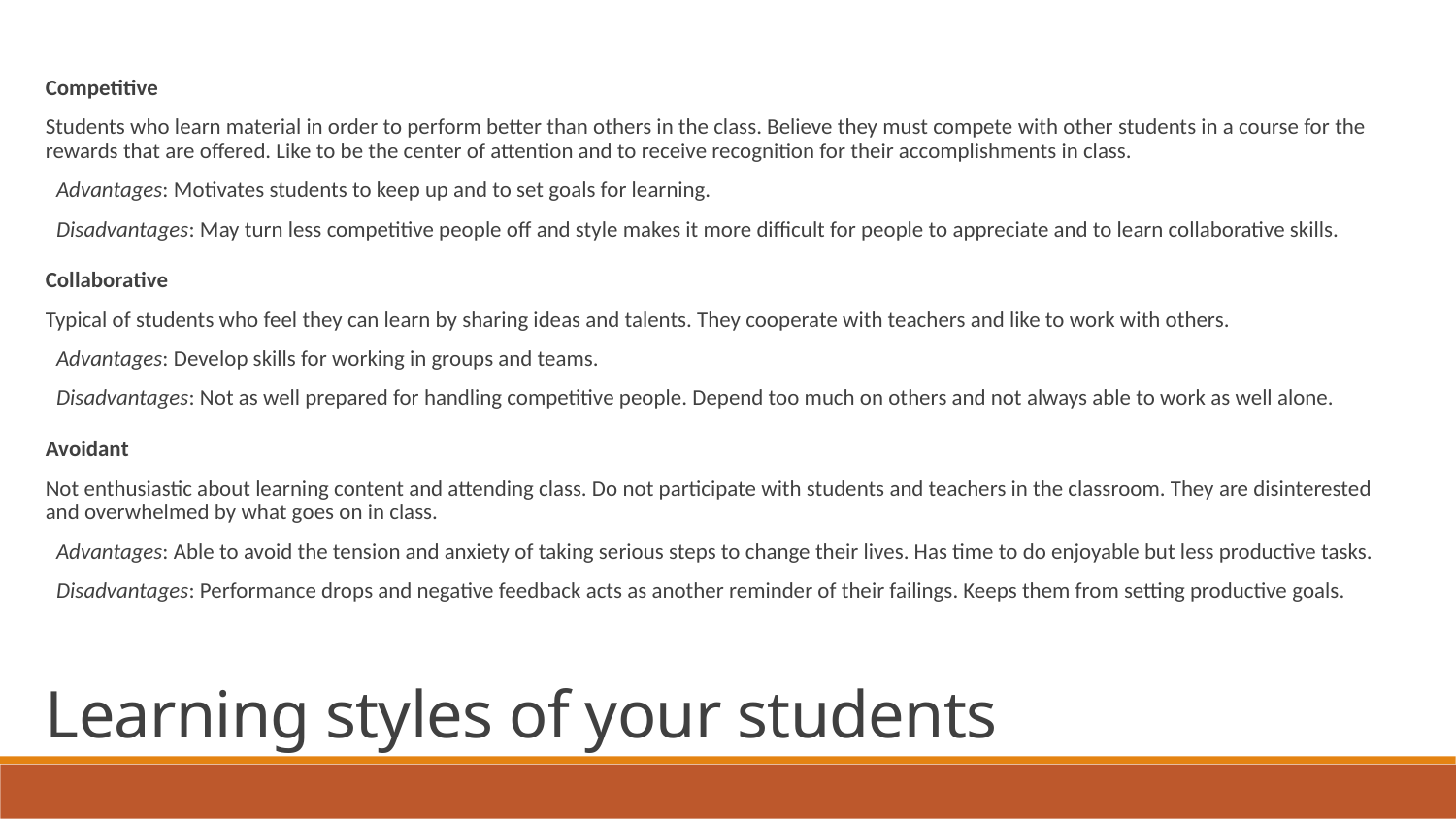

Competitive
Students who learn material in order to perform better than others in the class. Believe they must compete with other students in a course for the rewards that are offered. Like to be the center of attention and to receive recognition for their accomplishments in class.
Advantages: Motivates students to keep up and to set goals for learning.
Disadvantages: May turn less competitive people off and style makes it more difficult for people to appreciate and to learn collaborative skills.
Collaborative
Typical of students who feel they can learn by sharing ideas and talents. They cooperate with teachers and like to work with others.
Advantages: Develop skills for working in groups and teams.
Disadvantages: Not as well prepared for handling competitive people. Depend too much on others and not always able to work as well alone.
Avoidant
Not enthusiastic about learning content and attending class. Do not participate with students and teachers in the classroom. They are disinterested and overwhelmed by what goes on in class.
Advantages: Able to avoid the tension and anxiety of taking serious steps to change their lives. Has time to do enjoyable but less productive tasks.
Disadvantages: Performance drops and negative feedback acts as another reminder of their failings. Keeps them from setting productive goals.
Learning styles of your students

## Slide 11
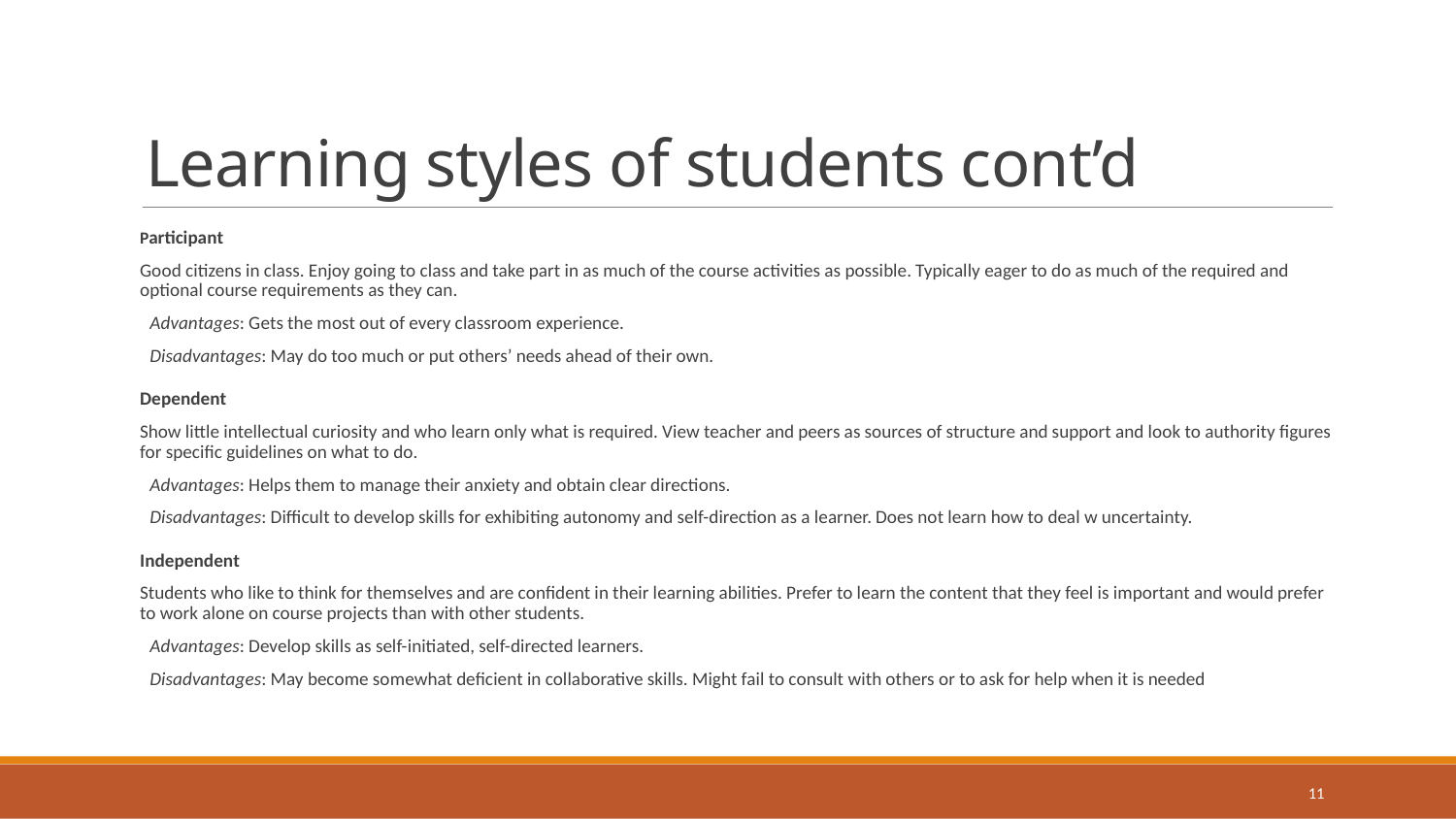

# Learning styles of students cont’d
Participant
Good citizens in class. Enjoy going to class and take part in as much of the course activities as possible. Typically eager to do as much of the required and optional course requirements as they can.
Advantages: Gets the most out of every classroom experience.
Disadvantages: May do too much or put others’ needs ahead of their own.
Dependent
Show little intellectual curiosity and who learn only what is required. View teacher and peers as sources of structure and support and look to authority figures for specific guidelines on what to do.
Advantages: Helps them to manage their anxiety and obtain clear directions.
Disadvantages: Difficult to develop skills for exhibiting autonomy and self-direction as a learner. Does not learn how to deal w uncertainty.
Independent
Students who like to think for themselves and are confident in their learning abilities. Prefer to learn the content that they feel is important and would prefer to work alone on course projects than with other students.
Advantages: Develop skills as self-initiated, self-directed learners.
Disadvantages: May become somewhat deficient in collaborative skills. Might fail to consult with others or to ask for help when it is needed
11

## Slide 12
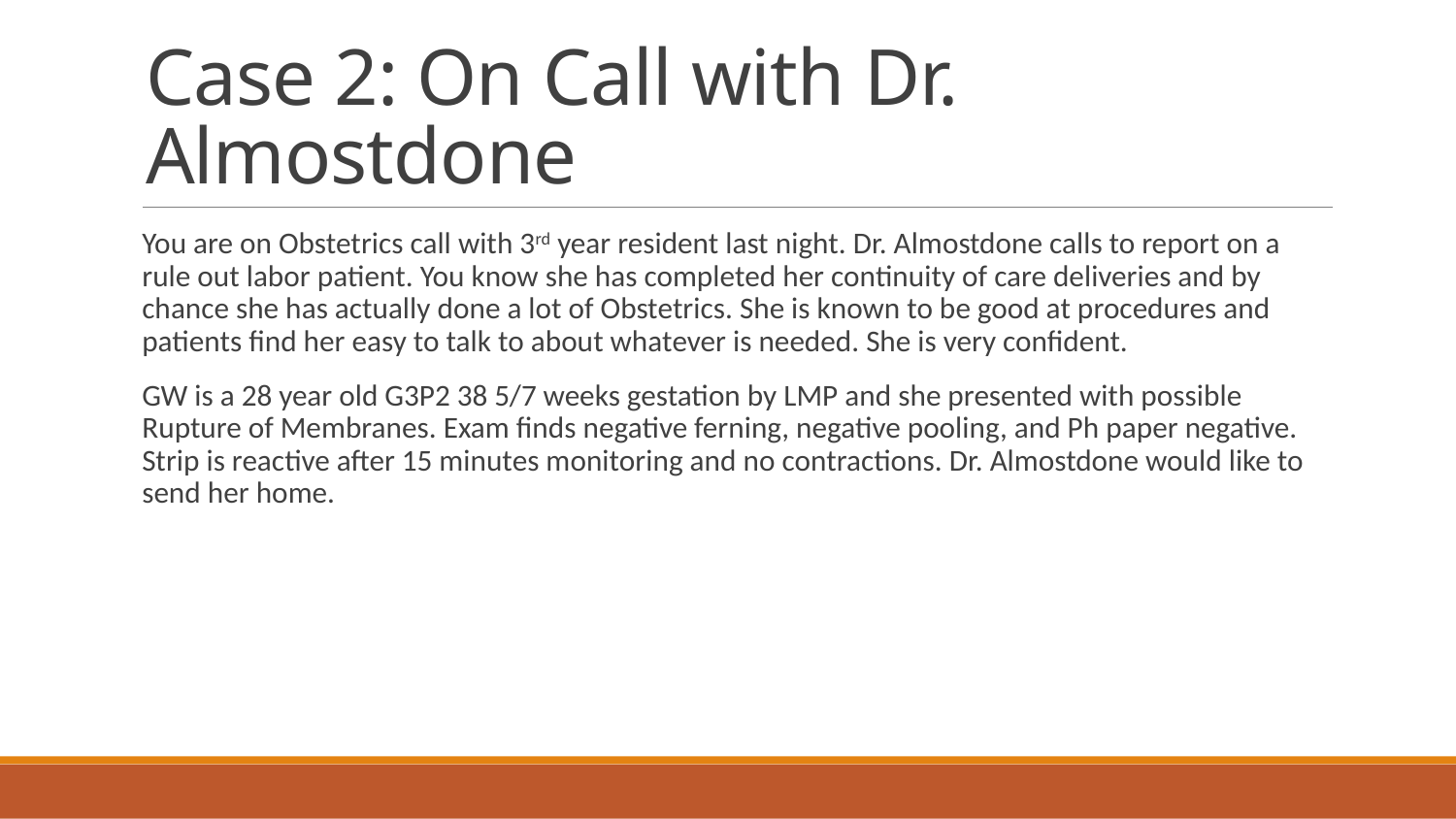

# Case 2: On Call with Dr. Almostdone
You are on Obstetrics call with 3rd year resident last night. Dr. Almostdone calls to report on a rule out labor patient. You know she has completed her continuity of care deliveries and by chance she has actually done a lot of Obstetrics. She is known to be good at procedures and patients find her easy to talk to about whatever is needed. She is very confident.
GW is a 28 year old G3P2 38 5/7 weeks gestation by LMP and she presented with possible Rupture of Membranes. Exam finds negative ferning, negative pooling, and Ph paper negative. Strip is reactive after 15 minutes monitoring and no contractions. Dr. Almostdone would like to send her home.

## Slide 13
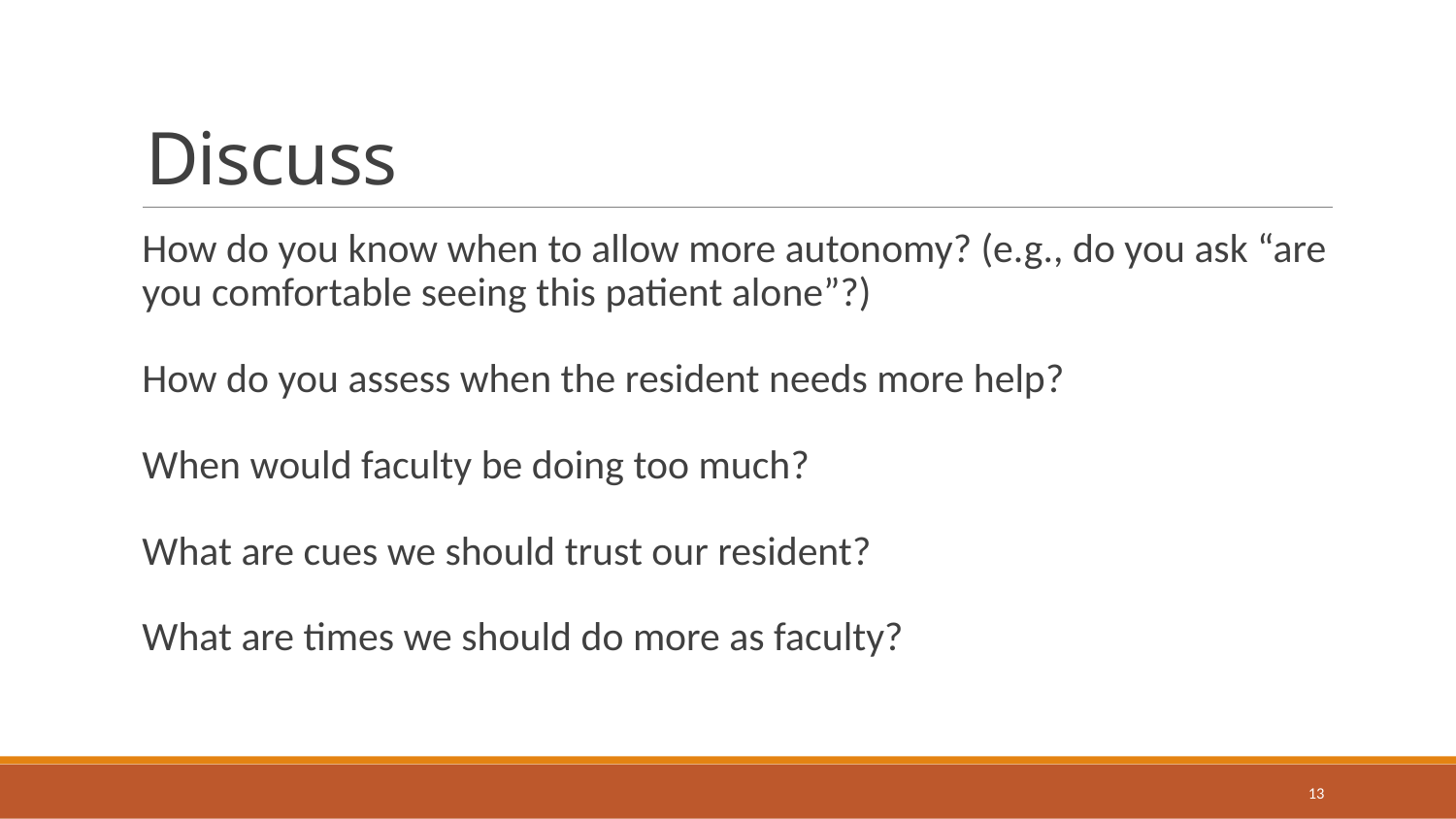

# Discuss
How do you know when to allow more autonomy? (e.g., do you ask “are you comfortable seeing this patient alone”?)
How do you assess when the resident needs more help?
When would faculty be doing too much?
What are cues we should trust our resident?
What are times we should do more as faculty?
13

## Slide 14
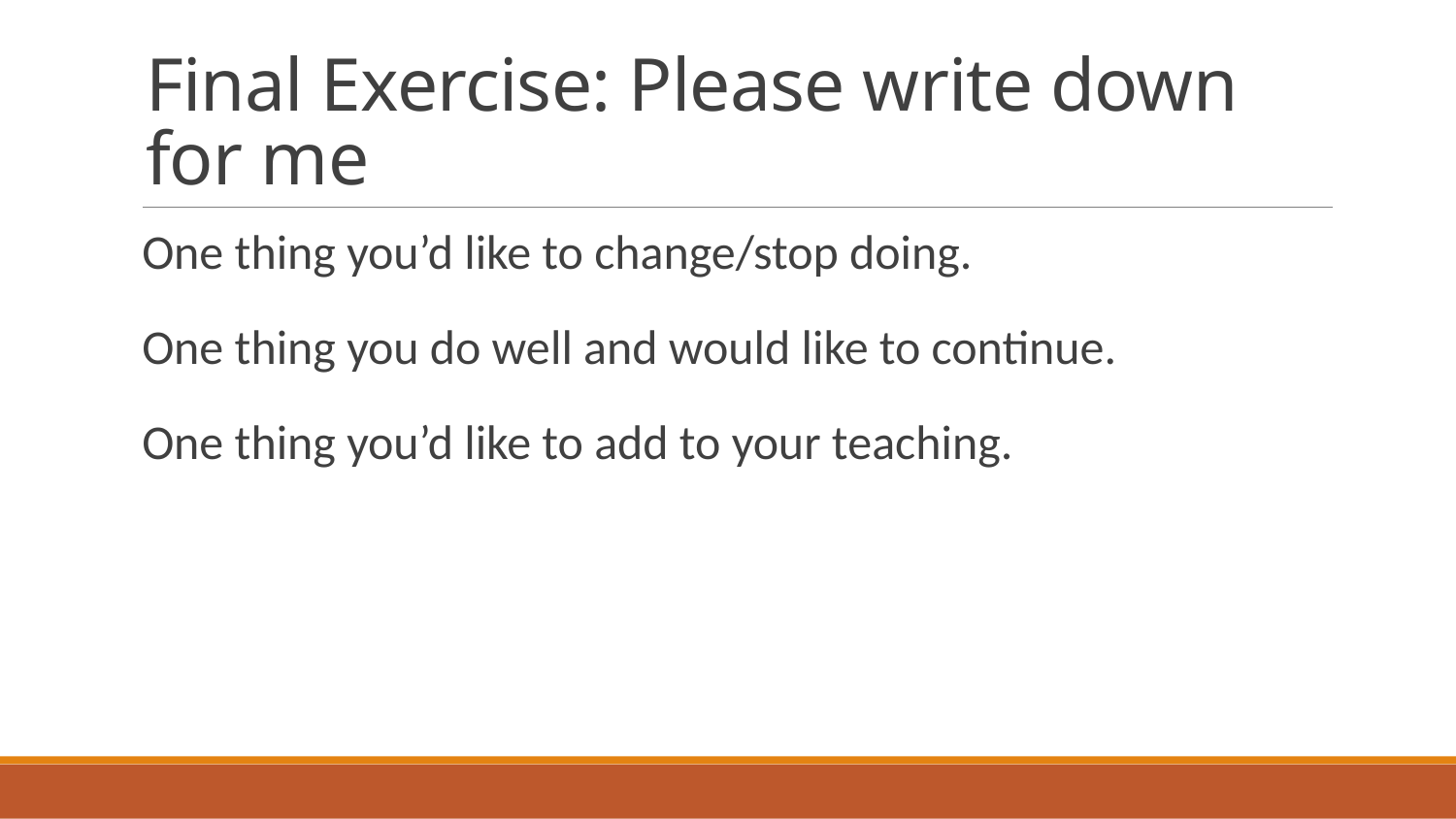

# Final Exercise: Please write down for me
One thing you’d like to change/stop doing.
One thing you do well and would like to continue.
One thing you’d like to add to your teaching.

## Slide 15
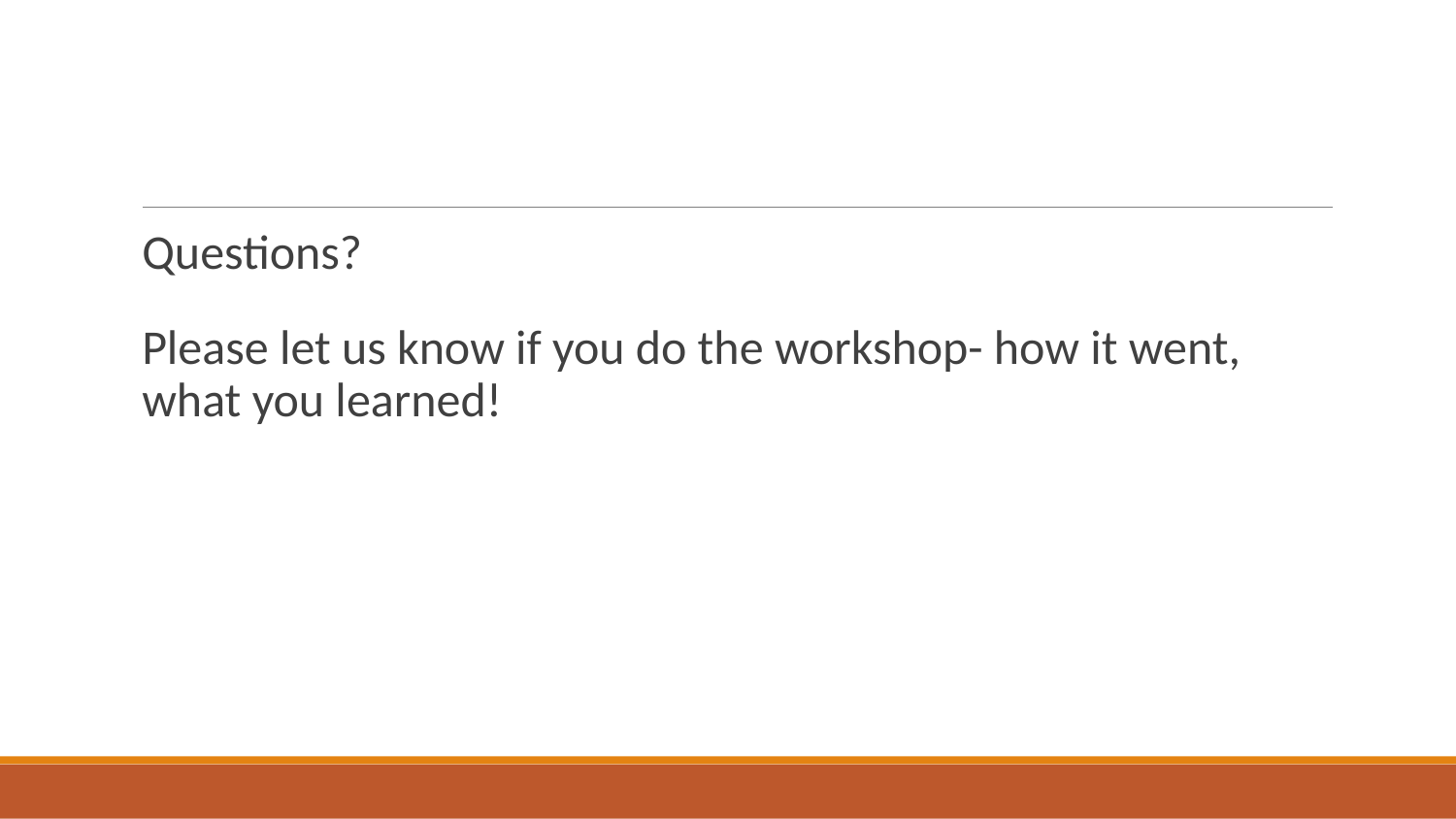

#
Questions?
Please let us know if you do the workshop- how it went, what you learned!

## Slide 16
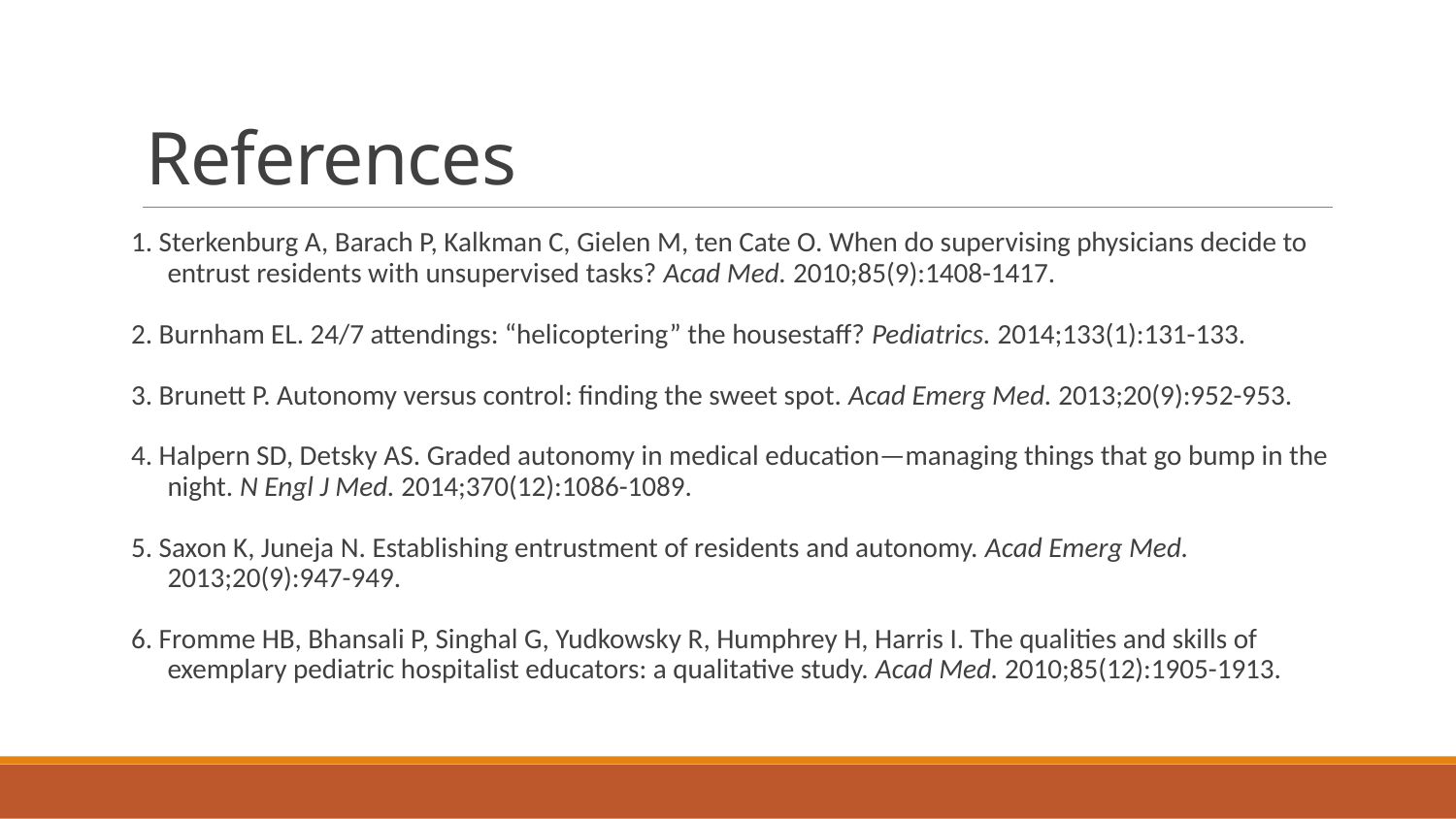

# References
1. Sterkenburg A, Barach P, Kalkman C, Gielen M, ten Cate O. When do supervising physicians decide to entrust residents with unsupervised tasks? Acad Med. 2010;85(9):1408-1417.
2. Burnham EL. 24/7 attendings: “helicoptering” the housestaff? Pediatrics. 2014;133(1):131-133.
3. Brunett P. Autonomy versus control: finding the sweet spot. Acad Emerg Med. 2013;20(9):952-953.
4. Halpern SD, Detsky AS. Graded autonomy in medical education—managing things that go bump in the night. N Engl J Med. 2014;370(12):1086-1089.
5. Saxon K, Juneja N. Establishing entrustment of residents and autonomy. Acad Emerg Med. 2013;20(9):947-949.
6. Fromme HB, Bhansali P, Singhal G, Yudkowsky R, Humphrey H, Harris I. The qualities and skills of exemplary pediatric hospitalist educators: a qualitative study. Acad Med. 2010;85(12):1905-1913.
