# Supplementary material for: Goldilocks and Entrustment: Finding the Amount of Learner Autonomy That's Just Right
Source: MedEdPORTAL. 2020 Oct 13;16:10987. doi: 10.15766/mep_2374-8265.10987 (PMC7566225; doi:10.15766/mep_2374-8265.10987)
Supplement: Supplementary file 1 — Goldilocks and Entrustment Workshop.pptxSelf-Evaluation Activity.docxSmall-Group Activity 1-Reflection.docxSmall-Group Activity 2-Comment Evaluation.docxCase 1-Dr. Newby.docxCase 2-Dr. Almostdone.docxAudience Commitment Form.docxPostworkshop Evaluation.docxAutonomy and Entrustment Facilitator Guide.docxAll Autonomy Workshop Handouts.docx [file mep_2374-8265.10987-s001.zip › C. Small-Group Activity 1-Reflection.docx]

1. Divide into groups of 3-4 members
2. Brainstorm examples from clinical experiences where faculty provided too much or too little autonomy and entrustment. For example:

- Faculty didn’t let you cut a suture when stitching because the tail would be too long or too short.
- Faculty didn’t let you put in a central line when you had never seen one before and the patient was clinically very unstable.

1. Ask volunteers to share examples from the small group discussion.
