# Supplementary material for: Goldilocks and Entrustment: Finding the Amount of Learner Autonomy That's Just Right
Source: MedEdPORTAL. 2020 Oct 13;16:10987. doi: 10.15766/mep_2374-8265.10987 (PMC7566225; doi:10.15766/mep_2374-8265.10987)
Supplement: Supplementary file 1 — Goldilocks and Entrustment Workshop.pptxSelf-Evaluation Activity.docxSmall-Group Activity 1-Reflection.docxSmall-Group Activity 2-Comment Evaluation.docxCase 1-Dr. Newby.docxCase 2-Dr. Almostdone.docxAudience Commitment Form.docxPostworkshop Evaluation.docxAutonomy and Entrustment Facilitator Guide.docxAll Autonomy Workshop Handouts.docx [file mep_2374-8265.10987-s001.zip › D. Small-Group Activity 2-Comment Evaluation.docx]

Randomly distribute the comments so that each small group has one set.

Distribute the comments so that each member in the small groups has a different set of comments.

**Comments 1**

- The service is as always a zoo and he/she is flexible enough to help us get through the day.
- He/she continues to be very hands-on with rounding and being a bit more in the background would be helpful to allow the senior to take more of a leadership role.
- I always would like more teaching but I was mostly trying to get my head above water so I’m not sure I would have retained more teaching.
- Gave some negative feedback in front of our entire team including nearby residents that probably could have waited until we were in a less public space.
- Tends to route everything through the senior. Sometimes I am asking for a faculty perspective when I ask a question. I know he/she is trying not to step on toes here but if I have to route everything through the senior (who is often busy with other things), I lose access to his/her insight and knowledge one on one.
- Has a habit of pontificating.
- I have serious misgivings about his/her medical knowledge. There were multiple times when I would ask him/her about what therapies to start and he/she would very confidently give an answer that turned out to be completely wrong and possibly dangerous. He/she does not work effectively on rounds and it is very difficult to get an actual definitive answer on what the plan will be for patients. I spent a lot of my time working with him/her reading about what he/she had told me to do in order to verify that it was medically sound.
- Should allow the residents more autonomy over their patients. Sometimes while rounding it seems as though he/she is not listening to the resident's presentation.
- Dominates the patient interaction in the room, which takes away from the residents’ ability to learn that skill.

**Comments 2**

- Is extremely approachable and is great at strategizing effective care plans for individual patients. Precepting patients with him/her is both educational and non-anxiety-provoking.
- Allows residents the appropriate amount of autonomy.
- Let’s me come to conclusions on my own. Provides me with the direction in thinking about clinic practice in the community in comparison to at the U.
- Great at showing exam skills and talking through the process.
- Has always been consistent in following up/closing the loop on patients care and willing to answer my questions on patients who I might not have even staffed with him/her.
- He/she has a knack for gently pointing out areas that need improvement and noting things I did well. Staffing in the clinic is very efficient and allows me to keep my clinic running on time. I always come away having learned something that day.
- Gives residents autonomy to perform their duties. Offers constructive feedback in a timely manner, sometimes even after a specific patient encounter.
- Always challenges me to go beyond my limited list of differentials and stresses on completing a thorough work up that is excellent for patient safety.
- Is so efficient and fast that sometimes it is hard to keep up with him/her.
- In appropriate situations, may present and discuss (or ask learner) literature related to clinical questions to continue to promote ongoing practice improvement in the learner.

**Comments 3**

- Patience! Incredibly knowledgeable and constantly sharing his/her medical knowledge. Always reading the latest research about our current clinical problems. I always have such an enjoyable week when he/she is on. I particularly enjoy his/her “I have a few silly questions” as a means to point out where you may have missed something on a patient.
- Present and collaborative instead of tyrannical.
- Is an excellent bedside teacher. He/she talks through his physical exam so that I am able to see areas which I am lacking or could improve.
- Allows autonomy with the appropriate level of guidance and support.
- Very willing to engage and ask me good questions about my patient care decisions.
- Great quick 30 seconds bullets points for learning. Straight and to the point. Encourages us to lead conversations with patients on rounds.
- Bedside teaching in the room with patient and family. Allows for independent decision making for residents. Asking me for direct feedback regularly and was very interested in adapting his/her own practice as a teacher.
- Having residents justify plans, but in a non-threatening way which can be very educational. Always prepared with good data.
- Encouraged us to commit to a diagnosis/plan- allowing for us to make mistakes in a safe environment. Very effective bedside teaching and teaching on rounds. So patient with the learner on the team. Through yet efficient on morning rounds.

**Comments 4**

- Gives the appropriate autonomy and ability for residents to grow on their own.
- Allows residents the appropriate level of autonomy and ownership over their own.
- Is great to work with on call. He/she is detailed and patient-centered yet allows resident autonomy in developing their plans.
- Teaches at appropriate times. Very organized and tries to ensure learning every day.
- Great to work with. Allows plenty of autonomy to residents.
- Created a great working environment. I felt very comfortable asking questions and also not knowing the answers to the teaching questions asked of me.
- I always appreciate that he/she is a steady educator and I know he/she will expect some forethought about a patient's assessment and plan but from there will continue to teach about and beyond. I learn a lot from him/her both in the content of what he/she teaches and by his example. It is also great that he/she provides printed articles!
- Wonderful during procedures. A good balance between allowing for independence and guidance when needed.
- One of my favorite faculty members to work with. Balances allowing us to make our own management decisions while keeping patient safety and best outcomes at the forefront.
- Allows the right amount of autonomy and supervision. He/she is down to earth and helps residents see the big picture.
