# Supplementary material for: Goldilocks and Entrustment: Finding the Amount of Learner Autonomy That's Just Right
Source: MedEdPORTAL. 2020 Oct 13;16:10987. doi: 10.15766/mep_2374-8265.10987 (PMC7566225; doi:10.15766/mep_2374-8265.10987)
Supplement: Supplementary file 1 — Goldilocks and Entrustment Workshop.pptxSelf-Evaluation Activity.docxSmall-Group Activity 1-Reflection.docxSmall-Group Activity 2-Comment Evaluation.docxCase 1-Dr. Newby.docxCase 2-Dr. Almostdone.docxAudience Commitment Form.docxPostworkshop Evaluation.docxAutonomy and Entrustment Facilitator Guide.docxAll Autonomy Workshop Handouts.docx [file mep_2374-8265.10987-s001.zip › E. Case 1-Dr. Newby.docx]

You are on call with Dr. Newby, a first year resident on night float in August of his intern year. You’ve never worked with Dr. Newby before. It’s 10:30PM when you bridge an admission from the ED. The patient is an 83 year old female resident of a local nursing home. She fell getting up from the table after dinner. She has sustained a hip fracture and the orthopedic service plans for a repair tomorrow. Because of her age and complicated medical history, ortho requested family medicine service admit her.

- The patient has a history of mild dementia, osteoarthritis with multiple joint replacements, diabetes type 2 with peripheral neuropathy, hypertension, Coronary artery disease, depression, CKD, and anemia of chronic disease. She’s on about 15 drugs (but that’s not the point of this exercise, so don’t worry about it).
- The ED resident reports, “Despite all that she’s comfortable and stable. Labs are stable. EKG is normal. Vitals are fine. She’s good for the floor.”
- The senior resident needs to stay in Labor and delivery due to an actively laboring patient.

**Faculty Physician Participants Discuss:**

How do you know when to allow more autonomy? (e.g., do you ask “are you comfortable seeing this patient alone”?)

How do you assess when the resident needs more help?

When would faculty be doing too much?

What are cues we should trust our resident?

What are times we should do more as faculty?
