# Supplementary material for: Goldilocks and Entrustment: Finding the Amount of Learner Autonomy That's Just Right
Source: MedEdPORTAL. 2020 Oct 13;16:10987. doi: 10.15766/mep_2374-8265.10987 (PMC7566225; doi:10.15766/mep_2374-8265.10987)
Supplement: Supplementary file 1 — Goldilocks and Entrustment Workshop.pptxSelf-Evaluation Activity.docxSmall-Group Activity 1-Reflection.docxSmall-Group Activity 2-Comment Evaluation.docxCase 1-Dr. Newby.docxCase 2-Dr. Almostdone.docxAudience Commitment Form.docxPostworkshop Evaluation.docxAutonomy and Entrustment Facilitator Guide.docxAll Autonomy Workshop Handouts.docx [file mep_2374-8265.10987-s001.zip › F. Case 2-Dr. Almostdone.docx]

- You are on Obstetrics call with a 3^rd^ year resident last night. Dr. Almostdone calls to report on a rule out labor patient. You know she has completed her continuity of care deliveries and by chance she has actually done a lot of Obstetrics. She is known to be good at procedures and patients find her easy to talk to about whatever is needed. She is very confident.
- GW is a 28 year old G3P2 38 5/7 weeks gestation by LMP and she presented with possible Rupture of Membranes. Exam finds negative ferning, negative pooling, and Ph paper negative. Strip is reactive after 15 minutes monitoring and no contractions. Dr. Almostdone would like to send her home.

**Faculty Physician Participants Discuss:**

How do you know when to allow more autonomy? (e.g., do you ask “are you comfortable seeing this patient alone”?)

How do you assess when the resident needs more help?

When would faculty be doing too much?

What are cues we should trust our resident?

What are times we should do more as faculty?
