# Supplementary material for: Goldilocks and Entrustment: Finding the Amount of Learner Autonomy That's Just Right
Source: MedEdPORTAL. 2020 Oct 13;16:10987. doi: 10.15766/mep_2374-8265.10987 (PMC7566225; doi:10.15766/mep_2374-8265.10987)
Supplement: Supplementary file 1 — Goldilocks and Entrustment Workshop.pptxSelf-Evaluation Activity.docxSmall-Group Activity 1-Reflection.docxSmall-Group Activity 2-Comment Evaluation.docxCase 1-Dr. Newby.docxCase 2-Dr. Almostdone.docxAudience Commitment Form.docxPostworkshop Evaluation.docxAutonomy and Entrustment Facilitator Guide.docxAll Autonomy Workshop Handouts.docx [file mep_2374-8265.10987-s001.zip › G. Audience Commitment Form.docx]

**Faculty Name:**

**Email Address:**

| **To promote the right amount of learner autonomy…** | |
| --- | --- |
| One thing I would **change** is: |  |
| One thing I would **continue** is: |  |
| One skill I will **add** is: |  |
