# Supplementary material for: Goldilocks and Entrustment: Finding the Amount of Learner Autonomy That's Just Right
Source: MedEdPORTAL. 2020 Oct 13;16:10987. doi: 10.15766/mep_2374-8265.10987 (PMC7566225; doi:10.15766/mep_2374-8265.10987)
Supplement: Supplementary file 1 — Goldilocks and Entrustment Workshop.pptxSelf-Evaluation Activity.docxSmall-Group Activity 1-Reflection.docxSmall-Group Activity 2-Comment Evaluation.docxCase 1-Dr. Newby.docxCase 2-Dr. Almostdone.docxAudience Commitment Form.docxPostworkshop Evaluation.docxAutonomy and Entrustment Facilitator Guide.docxAll Autonomy Workshop Handouts.docx [file mep_2374-8265.10987-s001.zip › H. Postworkshop Evaluation.docx]

| Rate: 1 = Not at all; 2 = Slightly; 3 = Somewhat; 4 = Very; 5 = Extremely | Your Score |
| --- | --- |
| Value/usefulness of this Autonomy Workshop to you |  |
| Your level of interest in this topic |  |
| Quality of presentation |  |
| How likely you are to apply what you learned today |  |
| Your OVERALL rating for this session |  |
