# Supplementary material for: Goldilocks and Entrustment: Finding the Amount of Learner Autonomy That's Just Right
Source: MedEdPORTAL. 2020 Oct 13;16:10987. doi: 10.15766/mep_2374-8265.10987 (PMC7566225; doi:10.15766/mep_2374-8265.10987)
Supplement: Supplementary file 1 — Goldilocks and Entrustment Workshop.pptxSelf-Evaluation Activity.docxSmall-Group Activity 1-Reflection.docxSmall-Group Activity 2-Comment Evaluation.docxCase 1-Dr. Newby.docxCase 2-Dr. Almostdone.docxAudience Commitment Form.docxPostworkshop Evaluation.docxAutonomy and Entrustment Facilitator Guide.docxAll Autonomy Workshop Handouts.docx [file mep_2374-8265.10987-s001.zip › I. Autonomy and Entrustment Facilitator Guide.docx]

**Appendix I: Autonomy and Entrustment Facilitator’s Guide/Talking Points**

**Material requirements**

Room with computer and projector, PowerPoint slides (Appendix A), 2 copies of Self-Evaluation Activity (Appendix B) for each attendee, 1 copy each of Appendix D, G, and H for each attendee, Small Group Exercise (Appendix C), Comment Evaluation Activity (Appendix D), Cases (Appendices E and F), Audience Commitment Form (Appendix G), and post-workshop evaluation (Appendix H). Prior to the workshop, a teaching style scale can be utilized.

**Process**

Prior to the Workshop (optional activity)

The Grasha-Reichmann Teaching Style Inventory scale can be provided via email for participants to assess their own teaching style.

Appendix D should be created from evaluation of faculty with multiple comments provided by residents. The comments should be cleaned of any identifying information so that they are anonymous. We utilized three months of comments, but any duration of time where there are adequate feedback comments to see evidence for the range of too controlling and too lax as well as just right precepting would be perfect. The comments included in Appendix D can be used if desired in addition or instead.

**Workshop**

Have PowerPoint open with copies of Appendix B, D, G and H for each attendee.

Introduce the workshop

2 minutes: Review Workshop Objectives (Appendix A, slide 2).

1 minute: Attendee Self-Evaluation—Slide 3

Ask participants to do a self-evaluation of the degree of autonomy they allowed learners (Appendix B). Ask each attendee to think about how much autonomy they think they give learners and mark it on the scale. This activity is for their own reflection. We do not collect this.

5 minutes: Small Group Exercise 1—Reflection

Divide participants into groups of 3 or 4 members. Have them come up with examples of educators they had met in their training and career who were too controlling or too laid back (Appendix C). Ask volunteer participants to share a few examples of what the small group brainstormed with the entire group. The purpose of the exercise is to get participants thinking of both sides of the continuum and recognizing that either too much or too little autonomy would be possible. It is to help encourage faculty insight.

10 minutes: Small Group Exercise 2—Comment Evaluation Activity

Distributed hard copies of anonymized comments from resident evaluations of faculty supervision to each small group (Appendix D). The groups should take time to alternate reading the comments out loud and determining whether they thought the comments were positive or negative. Additionally, prompt participants to consider whether the comments could have been made about them as a faculty member. This is to help provide insight into the situation for those who have less insight than they might need.

1 minute: Ask the faculty participants to complete the self-evaluation a second time (Appendix B). Instruct them to reflect on any changes in their self-evaluation since the beginning of the workshop. Have them consider the comments and how they may have not considered the degrees of autonomy others give and how residents assess this. Once again, explain that this activity is for their own reflection. We do not collect this.

10 minutes: Case 1 (Appendix E) presents a resident case to allow discussion about the appropriate amount of faculty involvement in the resident’s patient care. Review this case with discussion about how faculty could react with too much or too little autonomy and what cues the resident might provide to impact this. The case is complex and serves to highlight the importance of recognizing resident factors such as experience, previous demonstration of history taking, physical exam skills, clinical decision making and each of the resident issues can promote and prevent independence. This discussion is not to provide explicit strategies to assess resident readiness, but instead serves to highlight the variety of resident issues that could be at play. It also highlights the patient issues that need to be considered for when the faculty can allow independence by showing a very complex and perhaps tenuous patient that needs assessment prior to determining how much autonomy to allow. Finally, the case does not highlight the faculty personal factors such as risk, personality, or practice style. Overall, the discussion is to encourage faculty focus on the faculty role in entrustment.

5 minutes: If learners completed the Grasha-Reichmann Teaching Style inventory prior to the workshop, reviewing the it with the Teaching and Learning Styles in the PowerPoint presentation is beneficial. This discussion can allow the faculty physician to consider learning and teaching styles as they assess how they teach the learner and assess the learner to determine if they can allow independence and increased autonomy. This information highlights the faculty physician opportunities to impact resident physician learner autonomy further by considering the actual resident physician learning style prior to the second case discussion and is optional.

10 minutes: Case 2 (Appendix F) presents a second case to encourage discussion about the appropriate amount of faculty involvement in the resident’s patient care. This case suggests that the resident involved may have experience and may have demonstrated skills suggesting that a fairly high level of entrustment would be merited. On the other hand, because the resident is noted to be “very confident”, the case also highlights learner characteristics that could be an issue with allowing autonomy based on false impressions of learner knowledge. This should encourage discussion about how faculty could react with too much or too little autonomy and what cues the resident might provide to impact this.

5 minutes: The final exercise is to have each attendee commit to one thing to change, one thing to continue, and one thing to add to teaching. Ask the participants to write these on an index card and email them this information 3 weeks later to remind them to follow-up on this learning (Appendix G).

1 minute: Evaluations of the workshops should be completed at all the workshop presentations (Appendix H).
