# Supplementary material for: Goldilocks and Entrustment: Finding the Amount of Learner Autonomy That's Just Right
Source: MedEdPORTAL. 2020 Oct 13;16:10987. doi: 10.15766/mep_2374-8265.10987 (PMC7566225; doi:10.15766/mep_2374-8265.10987)
Supplement: Supplementary file 1 — Goldilocks and Entrustment Workshop.pptxSelf-Evaluation Activity.docxSmall-Group Activity 1-Reflection.docxSmall-Group Activity 2-Comment Evaluation.docxCase 1-Dr. Newby.docxCase 2-Dr. Almostdone.docxAudience Commitment Form.docxPostworkshop Evaluation.docxAutonomy and Entrustment Facilitator Guide.docxAll Autonomy Workshop Handouts.docx [file mep_2374-8265.10987-s001.zip › J. All Autonomy Workshop Handouts.docx]

**Appendix B: Self-Evaluation Activity**

**Self-Evaluation**

Please rate yourself on a scale of 1-10.

Degree of resident autonomy/independence given


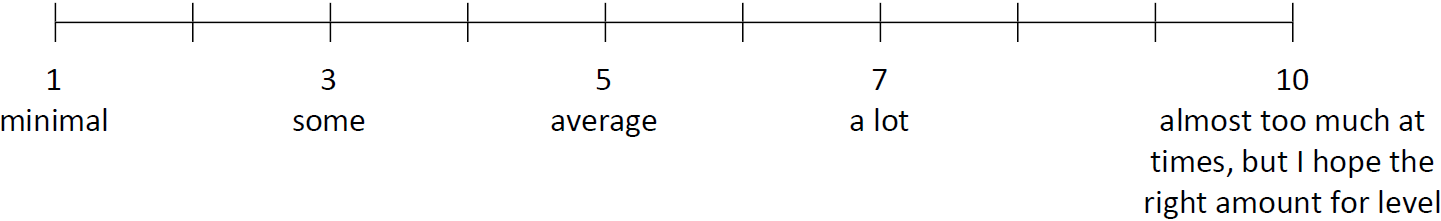


Amount of control I like when precepting


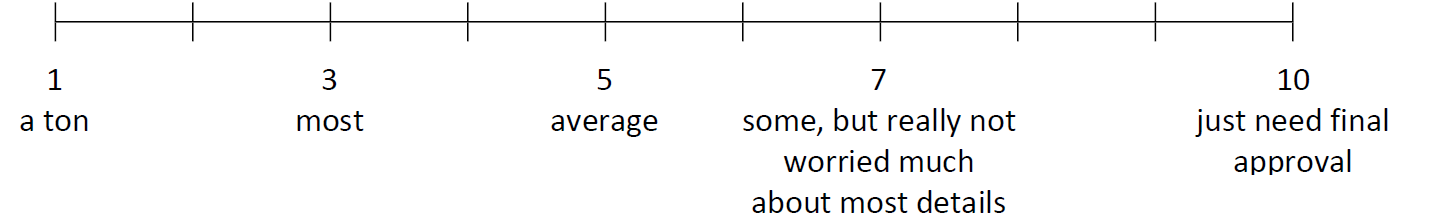


**Appendix C: Small Group Exercise 1—Reflection**

1. Divide into groups of 3-4 members
2. Brainstorm examples from clinical experiences where faculty provided too much or too little autonomy and entrustment. For example:

- Faculty didn’t let you cut a suture when stitching because the tail would be too long or too short.
- Faculty didn’t let you put in a central line when you had never seen one before and the patient was clinically very unstable.

1. Ask volunteers to share examples from the small group discussion.

**Appendix D: Small Group Exercise 2—Comment Evaluation Activity**

Randomly distribute the comments so that each small group has one set.

Distribute the comments so that each member in the small groups has a different set of comments.

**Comments 1**

- The service is as always a zoo and he/she is flexible enough to help us get through the day.
- He/she continues to be very hands-on with rounding and being a bit more in the background would be helpful to allow the senior to take more of a leadership role.
- I always would like more teaching but I was mostly trying to get my head above water so I’m not sure I would have retained more teaching.
- Gave some negative feedback in front of our entire team including nearby residents that probably could have waited until we were in a less public space.
- Tends to route everything through the senior. Sometimes I am asking for a faculty perspective when I ask a question. I know he/she is trying not to step on toes here but if I have to route everything through the senior (who is often busy with other things), I lose access to his/her insight and knowledge one on one.
- Has a habit of pontificating.
- I have serious misgivings about his/her medical knowledge. There were multiple times when I would ask him/her about what therapies to start and he/she would very confidently give an answer that turned out to be completely wrong and possibly dangerous. He/she does not work effectively on rounds and it is very difficult to get an actual definitive answer on what the plan will be for patients. I spent a lot of my time working with him/her reading about what he/she had told me to do in order to verify that it was medically sound.
- Should allow the residents more autonomy over their patients. Sometimes while rounding it seems as though he/she is not listening to the resident's presentation.
- Dominates the patient interaction in the room, which takes away from the residents’ ability to learn that skill.

**Comments 2**

- Is extremely approachable and is great at strategizing effective care plans for individual patients. Precepting patients with him/her is both educational and non-anxiety-provoking.
- Allows residents the appropriate amount of autonomy.
- Let’s me come to conclusions on my own. Provides me with the direction in thinking about clinic practice in the community in comparison to at the U.
- Great at showing exam skills and talking through the process.
- Has always been consistent in following up/closing the loop on patients care and willing to answer my questions on patients who I might not have even staffed with him/her.
- He/she has a knack for gently pointing out areas that need improvement and noting things I did well. Staffing in the clinic is very efficient and allows me to keep my clinic running on time. I always come away having learned something that day.
- Gives residents autonomy to perform their duties. Offers constructive feedback in a timely manner, sometimes even after a specific patient encounter.
- Always challenges me to go beyond my limited list of differentials and stresses on completing a thorough work up that is excellent for patient safety.
- Is so efficient and fast that sometimes it is hard to keep up with him/her.
- In appropriate situations, may present and discuss (or ask learner) literature related to clinical questions to continue to promote ongoing practice improvement in the learner.

**Comments 3**

- Patience! Incredibly knowledgeable and constantly sharing his/her medical knowledge. Always reading the latest research about our current clinical problems. I always have such an enjoyable week when he/she is on. I particularly enjoy his/her “I have a few silly questions” as a means to point out where you may have missed something on a patient.
- Present and collaborative instead of tyrannical.
- Is an excellent bedside teacher. He/she talks through his physical exam so that I am able to see areas which I am lacking or could improve.
- Allows autonomy with the appropriate level of guidance and support.
- Very willing to engage and ask me good questions about my patient care decisions.
- Great quick 30 seconds bullets points for learning. Straight and to the point. Encourages us to lead conversations with patients on rounds.
- Bedside teaching in the room with patient and family. Allows for independent decision making for residents. Asking me for direct feedback regularly and was very interested in adapting his/her own practice as a teacher.
- Having residents justify plans, but in a non-threatening way which can be very educational. Always prepared with good data.
- Encouraged us to commit to a diagnosis/plan- allowing for us to make mistakes in a safe environment. Very effective bedside teaching and teaching on rounds. So patient with the learner on the team. Through yet efficient on morning rounds.

**Comments 4**

- Gives the appropriate autonomy and ability for residents to grow on their own.
- Allows residents the appropriate level of autonomy and ownership over their own.
- Is great to work with on call. He/she is detailed and patient-centered yet allows resident autonomy in developing their plans.
- Teaches at appropriate times. Very organized and tries to ensure learning every day.
- Great to work with. Allows plenty of autonomy to residents.
- Created a great working environment. I felt very comfortable asking questions and also not knowing the answers to the teaching questions asked of me.
- I always appreciate that he/she is a steady educator and I know he/she will expect some forethought about a patient's assessment and plan but from there will continue to teach about and beyond. I learn a lot from him/her both in the content of what he/she teaches and by his example. It is also great that he/she provides printed articles!
- Wonderful during procedures. A good balance between allowing for independence and guidance when needed.
- One of my favorite faculty members to work with. Balances allowing us to make our own management decisions while keeping patient safety and best outcomes at the forefront.
- Allows the right amount of autonomy and supervision. He/she is down to earth and helps residents see the big picture.

**Appendix E: Case 1 On Call with Dr. Newby**

You are on call with Dr. Newby, a first year resident on night float in August of his intern year. You’ve never worked with Dr. Newby before. It’s 10:30PM when you bridge an admission from the ED. The patient is an 83 year old female resident of a local nursing home. She fell getting up from the table after dinner. She has sustained a hip fracture and the orthopedic service plans for a repair tomorrow. Because of her age and complicated medical history, ortho requested family medicine service admit her.

- The patient has a history of mild dementia, osteoarthritis with multiple joint replacements, diabetes type 2 with peripheral neuropathy, hypertension, Coronary artery disease, depression, CKD, and anemia of chronic disease. She’s on about 15 drugs (but that’s not the point of this exercise, so don’t worry about it).
- The ED resident reports, “Despite all that she’s comfortable and stable. Labs are stable. EKG is normal. Vitals are fine. She’s good for the floor.”
- The senior resident needs to stay in Labor and delivery due to an actively laboring patient.

**Faculty Physician Participants Discuss:**

How do you know when to allow more autonomy? (e.g., do you ask “are you comfortable seeing this patient alone”?)

How do you assess when the resident needs more help?

When would faculty be doing too much?

What are cues we should trust our resident?

What are times we should do more as faculty?

**Appendix F: Case 2 On Call with Dr. Almostdone**

- You are on Obstetrics call with 3^rd^ year resident last night. Dr. Almostdone calls to report on a rule out labor patient. You know she has completed her continuity of care deliveries and by chance she has actually done a lot of Obstetrics. She is known to be good at procedures and patients find her easy to talk to about whatever is needed. She is very confident.
- GW is a 28 year old G3P2 38 5/7 weeks gestation by LMP and she presented with possible Rupture of Membranes. Exam finds negative ferning, negative pooling, and Ph paper negative. Strip is reactive after 15 minutes monitoring and no contractions. Dr. Almostdone would like to send her home.

**Faculty Physician Participants Discuss:**

How do you know when to allow more autonomy? (e.g., do you ask “are you comfortable seeing this patient alone”?)

How do you assess when the resident needs more help?

When would faculty be doing too much?

What are cues we should trust our resident?

What are times we should do more as faculty?

**Appendix G: Audience Commitment Form**

**Faculty Name:**

**Email Address:**

| **To promote the right amount of learner autonomy…** | |
| --- | --- |
| One thing I would **change** is: |  |
| One thing I would **continue** is: |  |
| One skill I will **add** is: |  |

**Appendix H: Workshop Evaluation**

| Rate: 1 = Not at all; 2 = Slightly; 3 = Somewhat; 4 = Very; 5 = Extremely | Your Score |
| --- | --- |
| Value/usefulness of this session to you |  |
| Your level of interest in this topic |  |
| Quality of presentation |  |
| How likely you are to apply what you learned today |  |
| Your OVERALL rating for this session |  |
